# A Novel Intronic Circular RNA Antagonizes Influenza Virus by Absorbing a microRNA That Degrades CREBBP and Accelerating IFN-β Production

**DOI:** 10.1128/mBio.01017-21

**Published:** 2021-07-20

**Authors:** Zhiyuan Qu, Fei Meng, Jianzhong Shi, Guohua Deng, Xianying Zeng, Jinying Ge, Yanbing Li, Liling Liu, Pucheng Chen, Yongping Jiang, Chengjun Li, Hualan Chen

**Affiliations:** a State Key Laboratory of Veterinary Biotechnology, Harbin Veterinary Research Institute, Chinese Academy of Agricultural Sciences, Harbin, People’s Republic of China; The Peter Doherty Institute for Infection and Immunity

**Keywords:** influenza virus, antiviral agent, circular RNA

## Abstract

Virus-host interactions are complicated processes, and multiple cellular proteins promote or inhibit viral replication through different mechanisms. Recent progress has implicated circular RNAs (circRNAs) in cancer biology and progression; however, the role of circRNAs in viral infection remains largely unclear. Here, we detected 11,620 circRNAs in A549 cells and found that 411 of them were differentially expressed in influenza virus-infected A549 cells. We characterized a novel intronic circRNA, AIVR, that was upregulated in influenza virus-infected A549 cells and found that silencing of AIVR significantly promoted influenza virus replication in A549 cells. We further found that AIVR predominantly localizes in the cytoplasm and works as a microRNA (miRNA) sponge. One of the miRNAs absorbed by AIVR binds the mRNA of CREBBP, which is an important component of the large nucleoprotein complex interferon beta (IFN-β) enhanceosome that accelerates IFN-β production. AIVR overexpression significantly increased the mRNA and protein levels of IFN-β in the influenza virus-infected A549 cells. Therefore, the upregulation of AIVR is a cellular antiviral strategy, with AIVR exerting its antiviral effect by absorbing miRNA and promoting the expression of CREBBP to facilitate IFN-β production. Our study provides new insights into the roles of circRNAs in the cellular innate antiviral response.

## INTRODUCTION

Influenza A viruses are categorized into different subtypes according to the antigenicity of their surface glycoproteins, namely, hemagglutinin (HA) and neuraminidase (NA). Currently, 16 HA subtypes (H1 to H16) and 9 NA subtypes (N1 to N9) have been detected in avian species, and a few new subtypes (H17, H18, N10, and N11) have been detected in bats (1, 2). Influenza viruses of the H1N1, H2N2, and H3N2 subtypes have caused four human influenza pandemics since 1918, and the H1N1 and H3N2 viruses are still circulating in humans globally (3, 4). Some influenza viruses bearing the HA of the H5 and H7 subtypes are highly pathogenic for chickens and have caused numerous disease outbreaks in poultry around the world; moreover, the H5N1, H5N6, H7N7, and H7N9 viruses have also caused severe human infections and deaths in different countries (5). In addition, H9N2 and H10N8 viruses have also occasionally infected humans (6, 7). Therefore, influenza viruses continue to pose a threat to human and animal health.

Cellular factors, including proteins and noncoding RNAs, play roles in the virus life cycle, and identification of these host factors and their underlying mechanisms can provide important insights for the development of strategies to inhibit viral infection. A number of host proteins have been reported to interact with different influenza virus proteins to promote or inhibit viral replication at different stages of the virus life cycle (8–12). In comparison to cellular proteins, the effects of long noncoding RNAs on influenza virus replication are not well understood, although several linear long noncoding RNAs, including NRAV, NEAT1, BISPR, and CMPK2, have been recently reported to regulate the cellular antiviral response during influenza virus infection (13–16).

Circular RNAs (circRNAs), a new member of the long noncoding RNA families, have been identified in a variety of organisms, including plants, animals, and humans (17). CircRNAs are generated from pre-mRNA through the process of back-splicing and are categorized into exonic circRNAs, exon-intron circRNAs, intronic circRNAs, intergenic circRNAs, and antisense circRNAs according to their sequences. Exonic circRNAs are exclusively produced from exons and represent the largest group of circRNAs. The exon-intron circRNAs retain intronic sequences between the back-spliced exons (at least two), and the intronic circRNAs are generated only from introns. These intron-retaining circRNAs predominantly exist in the nucleus (18, 19). The microRNAs (miRNAs) are small noncoding RNAs of 20 to 24 nucleotides that regulate gene expression by repressing translation or directing sequence-specific degradation of complementary mRNA (20) or by targeting the promoter sequence and inducing gene expression (21, 22).

Previous studies suggest that the exonic circRNAs predominantly exist in the cytoplasm, and one of their functions is to work as an miRNA sponge and, thus, to counteract miRNA-mediated degradation of mRNAs (23–26). Zhang et al. reported that intronic circRNAs are abundant in the nucleus and have little enrichment for miRNA target sites, suggesting one possible function for intronic circRNAs as positive regulators of their parent coding genes (18). Accumulating data indicate that circRNAs perform multiple functions in a variety of cellular processes associated with human diseases, such as Alzheimer’s disease and cancer (27, 28); however, the roles of circRNAs in virus infection have been largely uninvestigated.

In this study, we investigated the cellular circRNA response upon influenza virus infection and found that 411 circRNAs were differentially expressed in the virus-infected cells. We identified a novel human intronic circRNA (we named AIVR) that antagonizes influenza virus replication, and we uncovered the underlying mechanism by which AIVR exerts its antiviral effect.

## RESULTS

### Identification of differentially expressed circRNAs in influenza virus-infected A549 cells.

**FIG 1:**
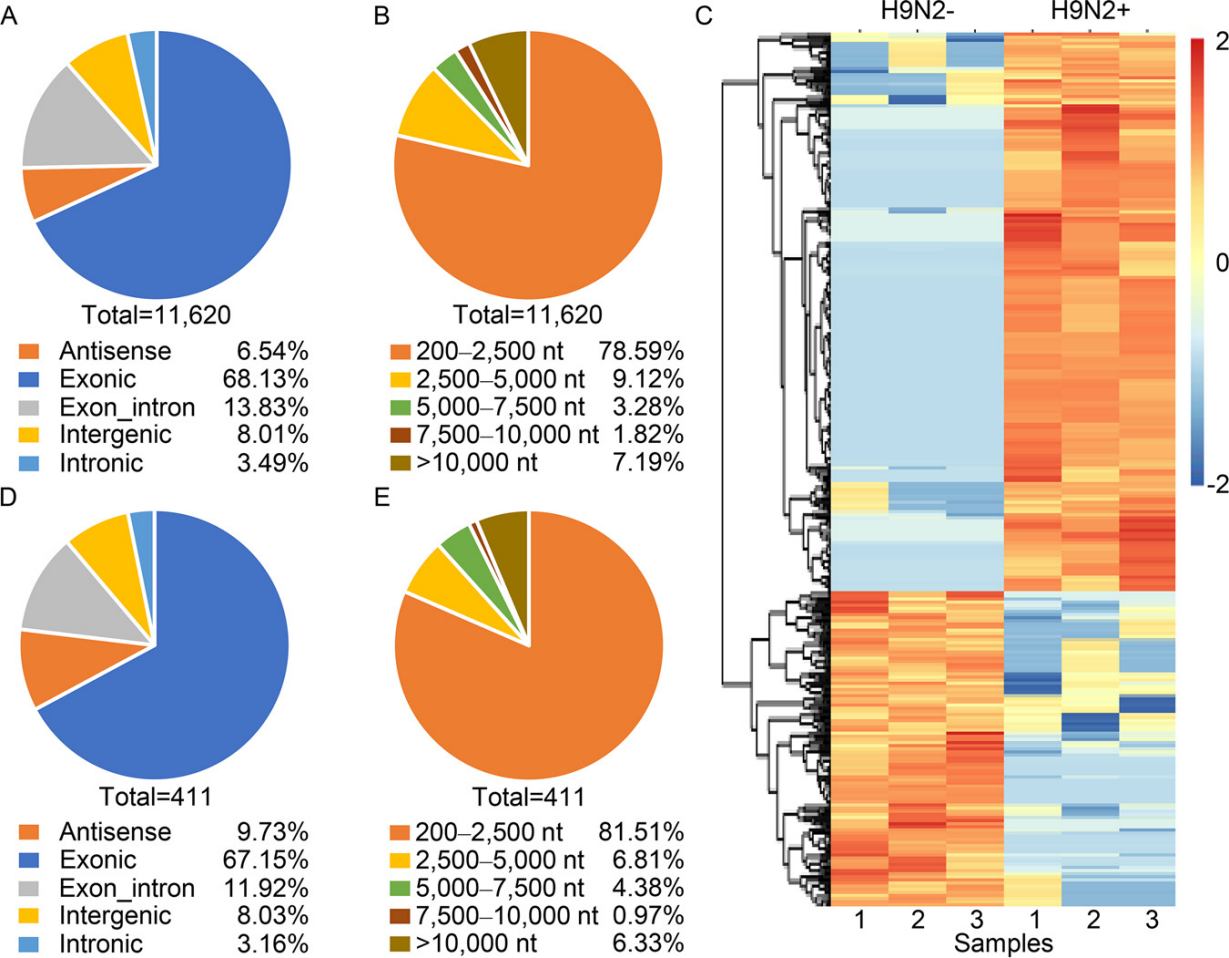
CircRNAs detected in H9N2 influenza virus-infected and uninfected A549 cells by RNA-Seq and bioinformatics. (A) Genomic origin of total circRNAs. (B) Length distribution of total circRNAs. (C) Heatmap of differentially expressed circRNAs. RNA-Seq analysis revealed 276 upregulated and 135 downregulated circRNAs in H9N2-infected A549 cells compared with H9N2-uninfected A549 cells (*n* = 3; |fold change|, >2; *P < *0.05). The RNA quantification is shown as centered and scaled log_2_ data in the heatmap. (D) Genomic origin of differentially expressed circRNAs. (E) Length distribution of differentially expressed circRNAs.

To investigate the differential expression of circRNAs in influenza virus-infected cells, human alveolar epithelial cells (A549) were mock infected or infected with an A/chicken/Jiangsu/C4258/2012 (H9N2) avian influenza virus (H9N2 virus). At 12 h postinfection (p.i.), total RNA was extracted from the cells, and the circRNAs, miRNAs, and mRNAs were deep sequenced and computationally analyzed. The bioinformatic analysis indicated that 11,620 distinct circRNAs (>200 nucleotides [nt]) were present in A549 cells (Fig. 1A and B), including 7,412 (63.8%) newly identified circRNAs and 4,208 (36.2%) circRNAs that have been reported by others in circBase (29). A total of 411 differentially expressed circRNAs, including 276 upregulated and 135 downregulated, were detected after the H9N2 virus infection (|fold change| of >2; *P < *0.05) (Fig. 1C). Among the total circRNAs, over 68% were generated from exons and only 3.49% from introns (Fig. 1A). The length of the circRNAs ranged from 200 to 91,766 nucleotides, and 78.59% of them were smaller than 2,500 nt (Fig. 1B). Similar distribution patterns were observed for the differentially expressed circRNAs (Fig. 1D and E).

**FIG 2:**
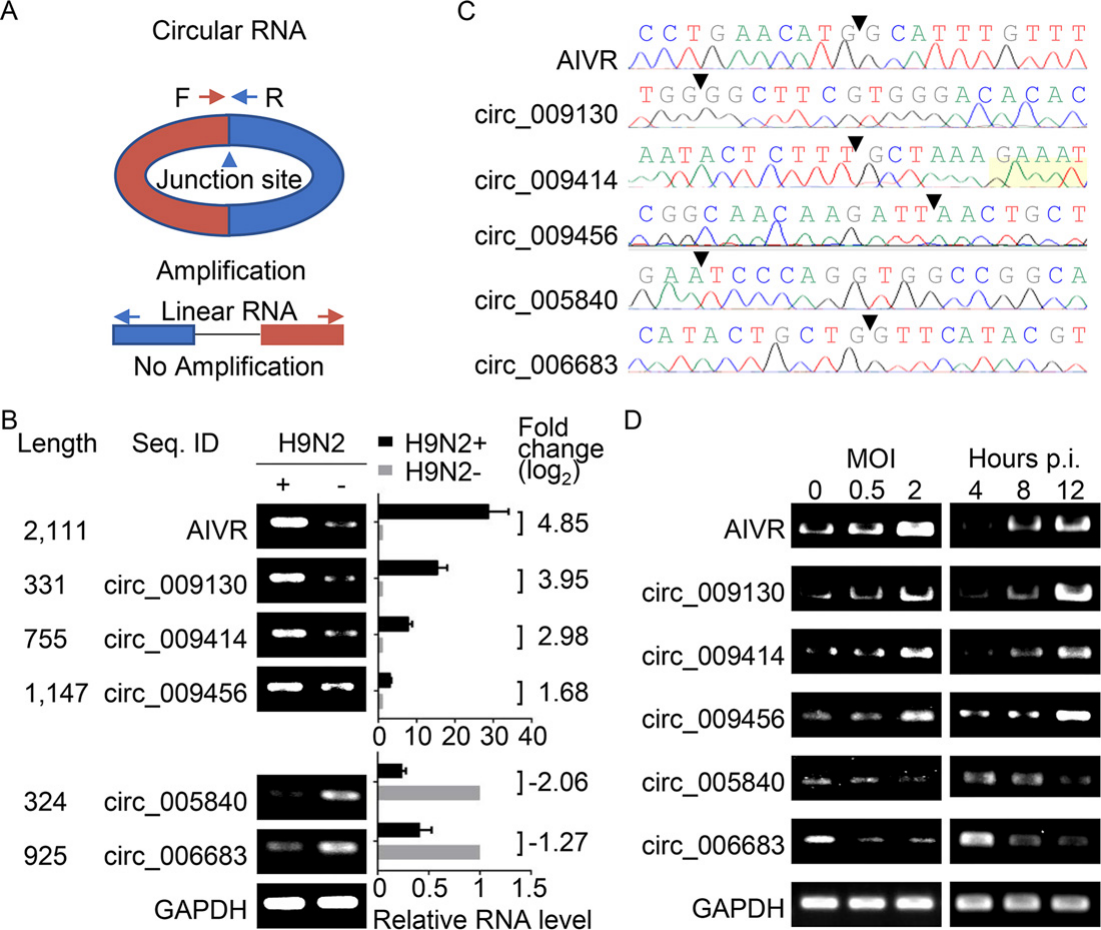
Validation of the circular nature of selected circRNAs. (A) Schematic diagram of the PCR primers. Expected products could be amplified only from circular RNAs and not from linear RNAs. (B) Validation of the differential expression of circRNAs upon influenza virus infection. Six circRNAs, ranging from 324 nt to 2,111 nt, were selected and quantified by qRT-PCR with primers designed to amplify the junction areas; data shown are the mean ± SD of three samples. (C) Junction sites of circRNAs identified by Sanger sequencing. The junction sites are indicated by arrowheads. (D) Expression level of the circRNAs in A549 cells infected with different doses of H9N2 virus or at different time points after virus infection.

The above prediction of circRNAs is based on transcriptome sequencing (RNA-Seq) and bioinformatics. However, many factors such as template switching, sequence homology, and degenerate sequences at exon boundaries may lead to false positive in such a prediction (30–32). We then selected six circRNAs that were abundant in A549 cells and highly differentially expressed after viral infection and verified their circular properties. Four upregulated circRNAs (AIVR, circ_009130, circ_009414, and circ_009456) and two downregulated circRNAs (circ_005840 and circ_006683) of A549 cells that were infected with or without H9N2 virus were quantified by reverse transcription-quantitative PCR (qRT-PCR) using a set of divergent primers that were designed to specifically amplify circRNAs (Fig. 2A; see Table S1 in the supplemental material). The differential expression of the six circRNAs in H9N2 virus-infected cells was confirmed by qRT-PCR (Fig. 2B), and the junction sites of these six circRNAs were analyzed by using Sanger sequencing (Fig. 2C).

10.1128/mBio.01017-21.1 TABLE S1 Sequences of primers, probes, siRNA, miRNA, and miRNA inhibitors used in this study^a^. a, Mutated sequences are underlined. Download Table S1, DOCX file, 0.04 MB. Copyright © 2021 Qu et al. 2021 Qu et al. https://creativecommons.org/licenses/by/4.0/ This content is distributed under the terms of the Creative Commons Attribution 4.0 International license.

The levels of the six differentially expressed circRNAs in A549 cells that were infected with different viral doses or that were collected at different time points after virus infection were evaluated. We found that the levels of the four upregulated circRNAs of the cells that were infected at an multiplicity of infection (MOI) of 2 with H9N2 virus were notably higher than those of the cells that were infected with the same virus but at an MOI of 0.5. The levels of the four upregulated circRNAs of the cells at 12 h p.i. were notably higher than those of the cells at 4 or 8 h p.i. with the H9N2 virus (MOI of 1). The levels of the two downregulated circRNAs of the cells that were infected at an MOI of 2 with H9N2 virus were notably lower than those of the cells that were infected at an MOI of 0.5 with H9N2 virus, and the levels of the two downregulated circRNAs of the cells at 12 h p.i. were notably lower than those of the cells at 4 or 8 h p.i. with the H9N2 virus (MOI of 1) (Fig. 2D). These results indicate that the expression levels of the circRNAs are related to viral dose and infection time.

### Characterization of a circRNA that antagonizes influenza virus replication.

**FIG 3:**
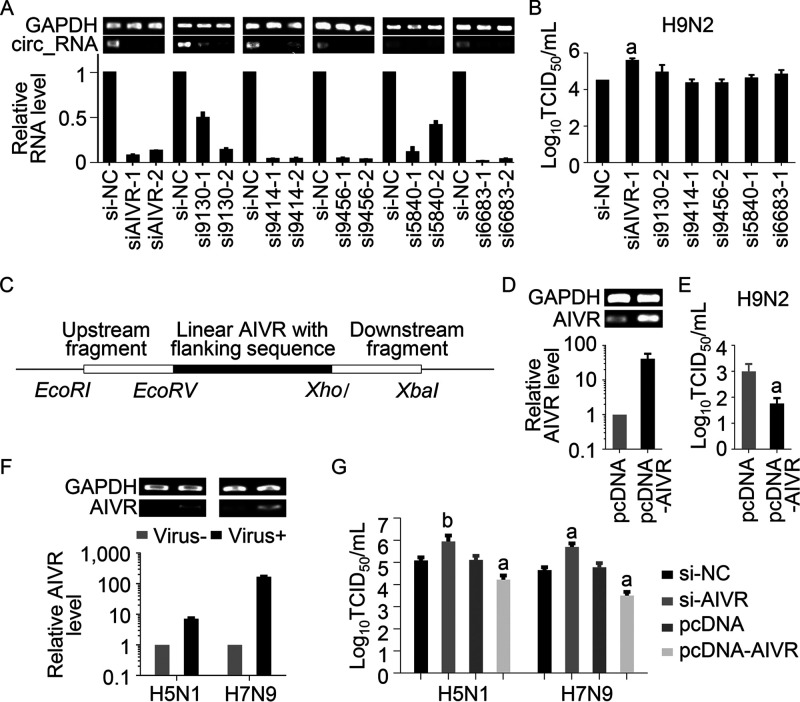
CircRNA AIVR inhibits the replication of different influenza viruses in A549 cells. (A) CircRNAs in A549 cells could be silenced by small interfering RNAs (siRNAs). A549 cells were transfected with different siRNAs or control siRNA (si-NC), and the level of different circRNAs was determined by qRT-PCR (*n* = 3); data shown are mean ± SD. (B) Silencing of AIVR promotes H9N2 virus replication. A549 cells were transfected with siRNA or si-NC, and the cells were infected 24 h later with H9N2 virus. Virus titers in supernatants were measured at 12 h postinfection. a, *P* value of <0.05 compared with the corresponding value of the si-NC-transfected cells. (C) Schematic illustration of the AIVR-overexpressing plasmid (pcDNA-AIVR) construction. The upstream fragment is amplified from the genomic sequence of intron 4 of MLLT3/AF9 (chr9, 20414651 to 20415428; hg38). The downstream fragment is the inverted upstream fragment. A linear AIVR sequence with 44-nt upstream flanking sequences and 84-nt downstream flanking sequences was amplified from genomic DNA of A549 cells. (D) Levels of circRNA AIVR in A549 cells. A549 cells were transfected with pcDNA-AIVR and pcDNA, respectively, and the levels of circRNA AIVR were determined by qRT-PCR 24 h posttransfection. (E) Overexpression of AIVR inhibited H9N2 replication. A549 cells were transfected with pcDNA-AIVR and pcDNA, respectively, and 24 h later, the cells were infected with H9N2 virus. Supernatants were harvested 24 h postinfection and viral titers were determined in MDCK cells. (F) AIVR was upregulated in A549 cells upon H5N1 or H7N9 virus infection. A549 cells were infected with H5N1 virus or H7N9 virus (MOI, 1), respectively, and the level of AIVR in the cells was measured 12 h after infection by qRT-PCR. (G) The effect of AIVR on the replication of H5N1 and H7N9 viruses in A549 cells. A549 cells were transfected with AIVR siRNA (si-AIVR), si-NC, pcDNA-AIVR, and pcDNA, and 24 h later, the cells were infected with H5N1 or H7N9 virus. Viral titers in supernatants were titrated 36 h postinfection. Data shown are means ± SD. a, *P* value of <0.01 compared with the corresponding values of the si-NC- or pcDNA-transfected cells. b, *P *value of <0.05 compared with the corresponding values of the si-NC- or pcDNA-transfected cells.

Our data indicate that hundreds of circRNAs are differentially expressed upon influenza virus infection; but do any of these circRNAs affect viral replication? To answer this question, we selected the four upregulated circRNAs and the two downregulated circRNAs that were validated above and investigated whether they affect influenza virus replication. We designed two small interfering RNAs (siRNAs) covering the junction site of each circRNA and tested their efficiency for circRNA silencing (Fig. 3A). The siRNAs that efficiently interfered with the circRNAs were then selected for further study. Twenty-four hours after the siRNA transfection, A549 cells were infected with H9N2 virus at an MOI of 1, and the supernatants were harvested at 12 h p.i. for viral titration in MDCK cells. Silencing of AIVR enhanced the viral replication by 12-fold, whereas silencing of the other five circRNAs had no notable effects on H9N2 virus replication (Fig. 3B).

To further confirm the antiviral effect of AIVR, we constructed a plasmid to overexpress AIVR (designated pcDNA-AIVR) and confirmed that pcDNA-AIVR-transfected A549 cells have increased levels of circRNA AIVR (Fig. 3C and D). We then compared the replication of H9N2 virus in A549 cells that were first transfected with a pcDNA3.1 plasmid control (pcDNA) or pcDNA-AIVR. The viral titers in the pcDNA-AIVR-transfected cells were 17-fold lower than those in the control cells (Fig. 3E). These results indicate that the upregulation of circRNA AIVR in virus-infected cells is a host antiviral strategy against H9N2 virus infection.

We next investigated whether the antiviral efficacy mediated by AIVR is applicable to other influenza virus subtypes. We found that circRNA AIVR levels also increased after the cells were infected with an H5N1 virus (A/Anhui/1/2005 strain) or an H7N9 virus (A/Anhui/1/2013 strain) that were isolated from human patients (33, 34) (Fig. 3F). The titers of the H5N1 and H7N9 viruses in the AIVR-silenced A549 cells were 8-fold and 11-fold higher, respectively, than those in the control cells, whereas the titers of the H5N1 and H7N9 viruses in the cells overexpressing AIVR were 8-fold and 19-fold lower, respectively, than those in the control A549 cells (Fig. 3G). These results indicate that AIVR upregulation is a cellular antiviral strategy against infection with different subtypes of influenza viruses.

### AIVR is an intronic circRNA that predominantly localizes to the cytoplasm and binds different miRNAs in A549 cells.

**FIG 4:**
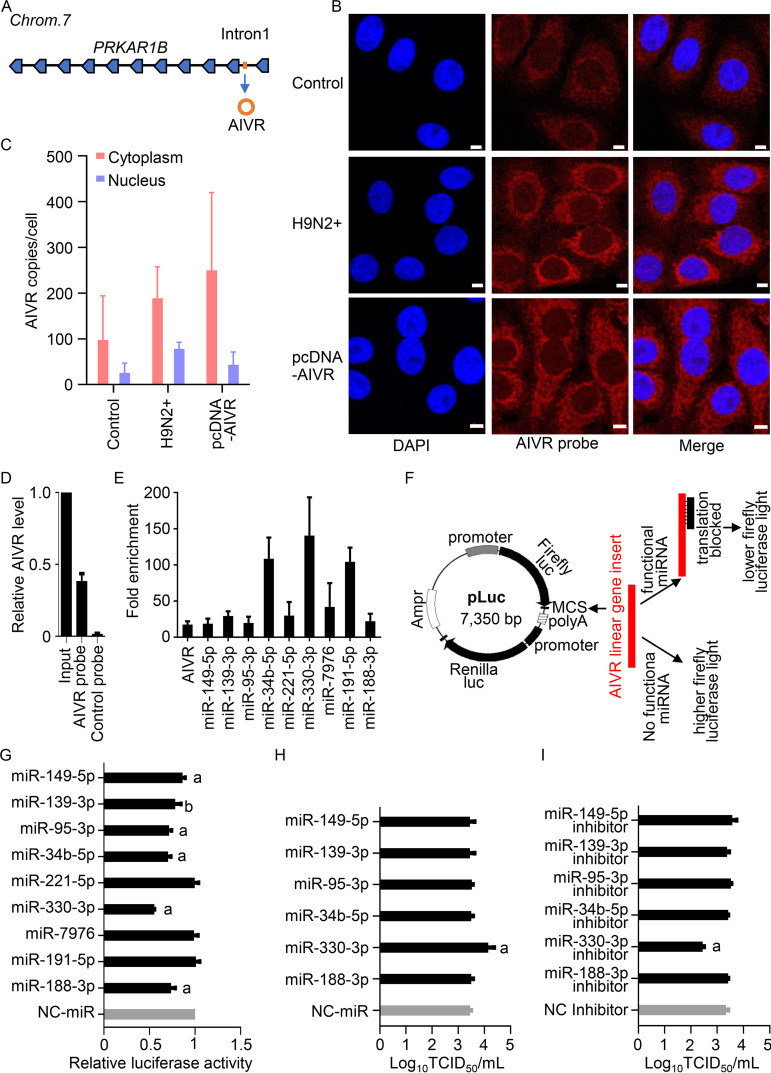
AIVR predominantly locates in the cytoplasm and binds to different miRNAs in A549 cells. (A) Schematic illustration of the genomic origin of AIVR. (B) Detection of AIVR in A549 cells by FISH. A549 cells were infected with H9N2 virus or transfected with pcDNA-AIVR, and AIVR in A549 cells was detected by an AIVR-specific FISH probe at 12 h after infection or 24 h after transfection. Nuclei were stained with DAPI. The white scale bar denotes 5 μm in all images. (C) AIVR copy numbers in the cytoplasm and nucleus of A549 cells were measured by qRT-PCR. (D) AIVR precipitation from AIVR-overexpressing A549 cells by using a specific biotin-labeled probe and a control probe. The relative copy numbers of AIVR pulled down by the probes were determined by qRT-PCR and were normalized to the value in the input. (E) AIVR absorbed miRNAs in A549 cells. The relative AIVR and miRNA levels pulled down by the probes were determined by qRT-PCR; data shown are values for the AIVR-specific probe normalized to those for the control probe. Only the nine miRNAs that were highly enriched (14- to 139-fold higher in the samples assessed by using the AIVR-specific probe than in those assessed with the control probe) are shown in the figure. (F) Schematic illustration of the mechanism of action of the pLuc-AIVR-wt vector. (G) Binding of different miRNAs to AIVR was confirmed by use of a luciferase reporter gene assay. HEK293T cells were cotransfected with pLuc-AIVR-wt vector and miRNA or NC-miR. The luciferase activity of the pLuc-AIVR-wt vector was normalized to the NC-miR transfection groups. a, *P* value of <0.01 compared with the corresponding values of the NC-miR group. b, *P* value of <0.05 compared with the corresponding values of the NC-miR group. (H and I) Effect of miRNA miR-330-3p on influenza virus replication. A549 cells were transfected with miRNA or NC-miR (H) or miRNA inhibitor or negative-control inhibitor (NC inhibitor) (I), and 24 h later, the cells were infected with H9N2 virus. Twenty-four hours after infection, the supernatants were harvested for virus titer determination in MDCK cells. a, *P* value of <0.01 compared with the corresponding values of the NC-miR group (H) or the NC inhibitor group (I).

Our sequence analysis indicates that the novel circRNA AIVR has 2,111 nucleotides. Genome Blat (http://genome.ucsc.edu/) results indicate that AIVR matches the nucleotides between positions 722754 and 724864 of chromosome 7 and overlaps with intron 1 of the protein kinase cAMP-dependent type I regulatory subunit beta (PRKAR1B) gene (Fig. 4A). Therefore, AIVR is a novel intronic circRNA.

Previous studies have shown that circRNAs derived from introns are abundant in the nucleus and could promote transcription of their host genes (18, 35, 36). We therefore investigated the cellular distribution of AIVR and asked whether it promotes the transcription of the PRKAR1B gene. Fluorescence *in situ* hybridization (FISH) analysis in A549 cells, H9N2 virus-infected A549 cells, and AIVR-overexpressing A549 cells revealed that AIVR predominantly localizes to the cytoplasm (Fig. 4B). AIVR copy numbers in the cytoplasm and nucleus of untreated A549 cells, H9N2 virus-infected A549 cells, and AIVR-overexpressing A549 cells were measured by qRT-PCR (Fig. 4C) and found to be consistent with the FISH analysis. Overexpression or silencing of AIVR did not affect the transcription of the PRKAR1B gene in A549 cells (see Fig. S1 in the supplemental material).

10.1128/mBio.01017-21.5 FIG S1 qRT-PCR results showed that an decreased or increased level of AIVR did not affect the cellular level of PRKAR1B in A549 cells. Download FIG S1, TIF file, 0.2 MB. Copyright © 2021 Qu et al. 2021 Qu et al. https://creativecommons.org/licenses/by/4.0/ This content is distributed under the terms of the Creative Commons Attribution 4.0 International license.

CircRNAs located in the cytoplasm have been reported to function as sponges for miRNAs (23, 24). We therefore investigated whether AIVR absorbs miRNAs. By using RNAhybrid miRNA target prediction tools (37), 206 different miRNA binding sites were predicted in AIVR, of which 35 are present in A549 cells (Table S2). We therefore designed a probe that specifically binds AIVR and a probe that does not bind AIVR (Fig. 4D) and used these probes to identify miRNAs that bind AIVR in the AIVR-overexpressing A549 cells. The miRNAs that bound AIVR were quantified by qRT-PCR with a set of primers that were specifically designed for the miRNAs (Table S1). The copy numbers of nine miRNAs were 14- to 139-fold higher in the samples assessed by using the AIVR-specific probe than in those examined with the control probe (Fig. 4E), whereas the copy numbers of the other 26 miRNAs in the samples assessed by using the AIVR-specific probe were less than 7-fold higher than those examined with the control probe (data not shown). To exclude any nonspecific binding in these experiments, we constructed a firefly luciferase reporter gene plasmid, pLuc-AIVR-wt, containing the full-length linear sequence of AIVR inserted between the luciferase gene and the SV40 late poly(A) signal of the pmirGLO plasmid (Promega, USA) (Fig. 4F), and transfected HEK293T cells with pLuc-AIVR-wt and each of the 9 miRNAs or a nonspecific control miRNA (NC-miR). The luciferase activity of the transfected cells was measured at 36 h posttransfection. As shown in Fig. 4G, compared with the luciferase activity in NC-miR-transfected cells, transfection of six miRNAs reduced the expression of the luciferase reporter gene by 14% to 45%, whereas transfection of the other three miRNAs did not affect the luciferase activity (Fig. 4G). These results demonstrate that AIVR does function as a miRNA sponge and binds to at least six miRNAs in A549 cells.

10.1128/mBio.01017-21.2 TABLE S2 The microRNAs (miRNAs) in A549 cells that were predicted to bind circRNA AIVR by RNAhybrid software. Download Table S2, DOCX file, 0.03 MB. Copyright © 2021 Qu et al. 2021 Qu et al. https://creativecommons.org/licenses/by/4.0/ This content is distributed under the terms of the Creative Commons Attribution 4.0 International license.

To investigate whether any of these six miRNAs absorbed by AIVR affect influenza virus infection, we transfected A549 cells with each of the six miRNAs or a NC-miR and then infected the cells at an MOI of 0.01 with the H9N2 virus. Twenty-four hours later, the supernatants were collected to determine viral titer. The viral titer in the hsa-miR-330-3p (miR-330-3p)-transfected cells was 5-fold higher than that in the NC-miR-transfected cells, whereas the viral titers in the other five miRNA-transfected cells were similar to those in the NC-miR-transfected cells (Fig. 4H). We confirmed these results by testing viral replication in A549 cells that were transfected with each of the miRNA-specific inhibitors or a control inhibitor (NC inhibitor); the viral titer in the cells transfected with the miR-330-3p inhibitor was about 8-fold lower than that in the cells transfected with the NC inhibitor, whereas the viral titers in the cells transfected with the inhibitors of the other five miRNAs were similar to those in the cells transfected with the NC inhibitor (Fig. 4I). These results indicate that the miRNA miR-330-3p enhances influenza replication in A549 cells.

### The miRNA miR-330-3p and circRNA AIVR bind to each other but do not degrade each other.

**FIG 5:**
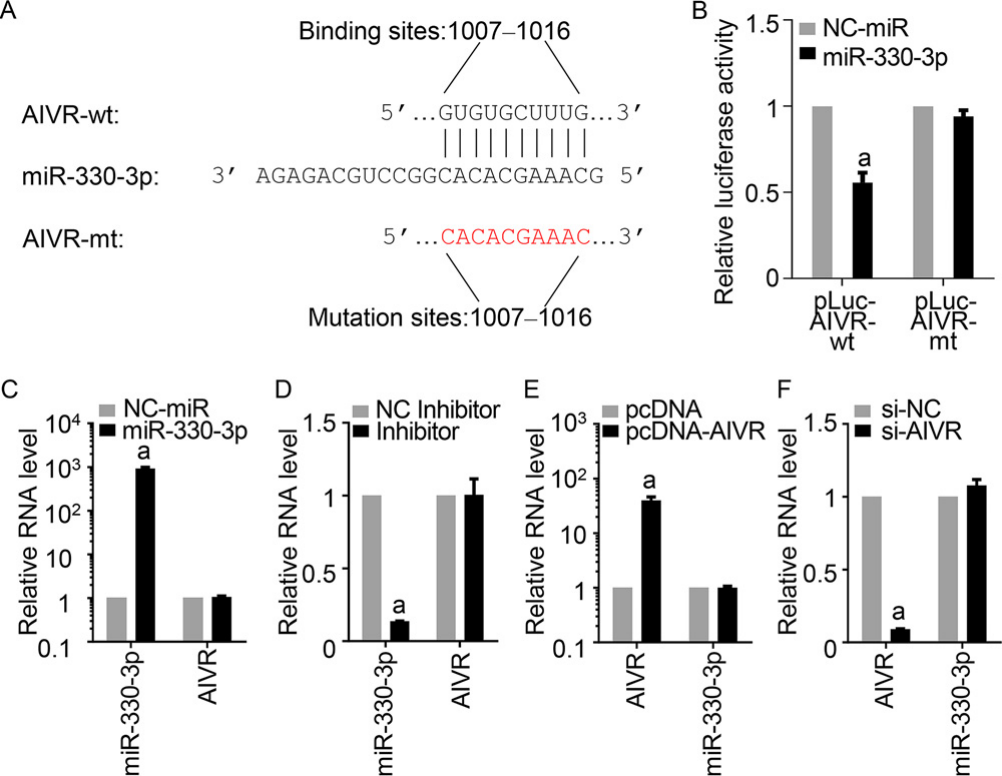
Interaction of circRNA AIVR and miRNA miR-330-3p. (A) Predicted binding site of miR-330-3p in AIVR. The predicted binding site sequences in AIVR were mutated in the plasmid pLuc-AIVR-mt as indicated in red. (B) miR-330-3p affects the luciferase activity of pLuc-AIVR-wt but not that of pLuc-AIVR-mt. HEK293T cells were transfected with miR-330-3p or NC-miR together with pLuc-AIVR-wt or pLuc-AIVR-mt. At 36 h posttransfection, a luciferase reporter gene assay was performed to measure luciferase activity. (C to F) AIVR and miR-330-3p cannot digest each other. qRT-PCR results showed that the increased (C) or decreased level (D) of miR-330-3p did not affect the cellular level of AIVR in A549 cells and that the increased (E) or decreased level (F) of AIVR did not affect the cellular level of miR-330-3p in A549 cells. The relative mRNA levels were determined by qRT-PCR. a, *P* value of <0.01 compared with the corresponding values of the control groups.

There are 23 nucleotides in miR-330-3p, and 10 nucleotides near its 5′ end match nucleotides 1007 to 1016 of circRNA AIVR (Fig. 5A). To determine whether this area of AIVR is the miRNA miR-330-3p binding site, we mutated these 10 nucleotides of circRNA AIVR in pLuc-AIVR-wt and designated this new plasmid pLuc-AIVR-mt (Fig. 5A). HEK293T cells were cotransfected with pLuc-AIVR-wt and miRNA miR-330-3p, pLuc-AIVR-wt and NC-miR, pLuc-AIVR-mt and miRNA miR-330-3p, or pLuc-AIVR-mt and NC-miR, and the luciferase activity of the transfected cells was measured at 36 h posttransfection. The luciferase activity in the cells transfected with pLuc-AIVR-wt and miRNA miR-330-3p was reduced by 45% compared with that in the cells transfected with pLuc-AIVR-wt and NC-miR, whereas the luciferase activity was comparable in the cells transfected with pLuc-AIVR-mt and miRNA miR-330-3p and the cells transfected with pLuc-AIVR-mt and NC-miR (Fig. 5B).

These results indicate that upregulated circRNA AIVR may exert its antiviral function by absorbing miRNA miR-330-3p; therefore, we investigated whether the two RNAs degraded each other. The copy numbers of circRNA AIVR in the miRNA miR-330-3p-transfected A549 cells were comparable with that in the NC-miR-transfected A549 cells (Fig. 5C). Similarly, circRNA AIVR levels were similar in the miRNA miR-330-3p-inhibited cells and the NC inhibitor-transfected cells (Fig. 5D). We also investigated the miRNA miR-330-3p levels in the AIVR-overexpressing and AIVR-silenced cells and found that the miRNA miR-330-3p levels were not affected by the levels of AIVR (Fig. 5E and F). These results indicate that AIVR and miR-330-3p bind to each other but do not degrade each other.

### The miRNA miR-330-3p interacts with different mRNAs.

Studies show that miRNA binds mRNA and affects the expression of related proteins through different mechanisms (20–22, 38, 39). Our data indicate that miRNA miR-330-3p promotes influenza virus replication, suggesting that miRNA miR-330-3p may interact with the mRNAs of certain proteins that directly or indirectly inhibit influenza virus replication. We therefore compared the mRNAs of H9N2 virus-infected A549 cells and uninfected A549 cells and found 5,031 upregulated mRNAs and 2,555 downregulated mRNAs (|fold change| of >2; false discovery rate value of <0.05) in the virus-infected cells (see Fig. S2 in the supplemental material). Among these genes, 57 upregulated genes have been linked to influenza virus infection by Kyoto Encyclopedia of Genes and Genomes (KEGG) pathway analysis (Table S3).

10.1128/mBio.01017-21.3 TABLE S3 Differentially expressed genes of influenza virus infection pathway and their interaction with miRNA miR-330-3p predicted by miRanda. Download Table S3, DOCX file, 0.03 MB. Copyright © 2021 Qu et al. 2021 Qu et al. https://creativecommons.org/licenses/by/4.0/ This content is distributed under the terms of the Creative Commons Attribution 4.0 International license.

10.1128/mBio.01017-21.6 FIG S2 The volcano plot of differentially expressed mRNA upon influenza A virus infection in A549 cells. The green and red points represent downregulated and upregulated mRNAs, respectively, in A549 cells upon H9N2 virus infection [|fold change|, >2; false discovery rate value, <0.05]. The orange points represent mRNAs with |fold change| of <2 and false discovery rate value of <0.05, and gray points represent mRNAs with false discovery rate value of >0.05 in the H9N2-infected A549 cells compared with uninfected A549 cells. The x axis represents a log_2_ ratio of mRNA expression levels between infected and uninfected A549 cells. The *y* axis represents the false discovery rate value (−log_10_ transformed) of mRNA. Download FIG S2, TIF file, 0.3 MB. Copyright © 2021 Qu et al. 2021 Qu et al. https://creativecommons.org/licenses/by/4.0/ This content is distributed under the terms of the Creative Commons Attribution 4.0 International license.

**FIG 6:**
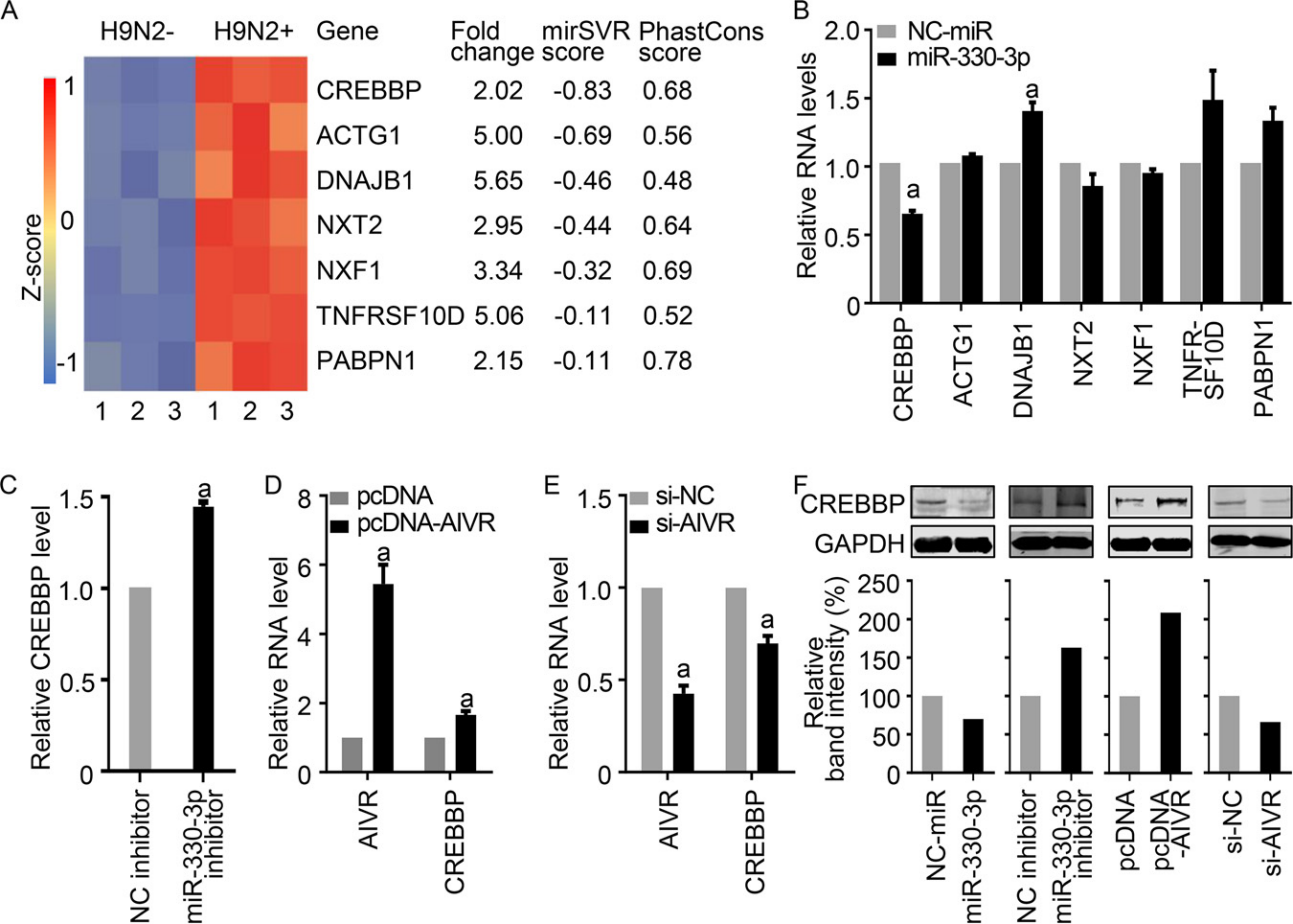
Identification of mRNAs in A549 cells that may be affected by miR-330-3p. (A) Seven differentially expressed mRNAs in H9N2 virus-infected A549 cells that may interact with miR-330-3p were predicted by miRanda algorithm (40). (B) Changes in the seven predicted mRNAs in miR-330-3p-transfected A549 cells. (C) Changes in the mRNA level of CREBBP in miR-330-3p-silenced A549 cells. (D and E) The mRNA levels of CREBBP in AIVR-overexpressing (D) or AIVR-silenced (E) A549 cells. (F) Western blotting of the protein level of CREBBP in miR-330-3p- and AIVR-overexpressing or -silenced A549 cells. The relative mRNA levels in B to E were determined by qRT-PCR. a, *P *value of <0.01 compared with the corresponding values of the control groups.

The interactions between miRNAs and mRNAs can be predicted by using the miRanda prediction algorithm (40). In such an analysis, if the miRNA and mRNA have an mirSVR score of less than −0.1 and a PhastCons score of greater than 0, the miRNA and mRNA have the potential to interact. We therefore predicted the interactions between miRNA miR-330-3p and the 57 mRNAs listed in Table S3 in the supplemental material by using the miRanda prediction algorithm and found that only 7 genes met both criteria (Fig. 6A; Table S3), suggesting that these 7 mRNAs may interact with miRNA miR-330-3p.

To test our prediction, we investigated whether the levels of these mRNAs are affected in A549 cells transfected with miRNA miR-330-3p. The mRNA levels of DNAJB1, TNFRSF10D, and PABPN1 in miRNA miR-330-3p-transfected cells were significantly or notably higher than those in NC-miR transfected cells. The mRNA levels of the other three proteins in the miRNA miR-330-3p-transfected cells were comparable with those in the NC-miR-transfected cells, whereas the mRNA level of CREBBP in the miRNA miR-330-3p-transfected cells was significantly lower than that in the NC-miR-transfected cells (Fig. 6B). We further confirmed that the mRNA level of CREBBP in the miRNA miR-330-3p-silenced cells was significantly higher than that in the NC-inhibitor-transfected cells (Fig. 6C).

We then investigated the mRNA levels of CREBBP in AIVR-overexpressing or -silenced A549 cells. The mRNA level of CREBBP in the AIVR-overexpressing cells was significantly higher than that in the control cells (Fig. 6D), and the mRNA level of CREBBP was significantly reduced when AIVR was silenced (Fig. 6E). These results indicate that AIVR positively affects the RNA level of CREBBP.

We next investigated the protein levels of CREBBP in cells with different miR-330-3p or AIVR levels and found that the protein levels of CREBBP in the miR-330-3p-transfected A549 cells and AIVR-silenced A549 cells were notably lower than those in their control cells and that the protein levels of CREBBP in the miR-330-3p-silenced A549 cells and AIVR-overexpressing A549 cells were notably higher than those in their control cells (Fig. 6F). These results indicate that in A549 cells containing different levels of miR-330-3p or AIVR, changes in CREBBP protein levels were consistent with its mRNA levels and that upregulation of AIVR promotes CREBBP expression.

### The circRNA AIVR absorbs the miRNA miR-330-3p, promotes CREBBP expression, and thereby facilitates IFN-β production.

**FIG 7:**
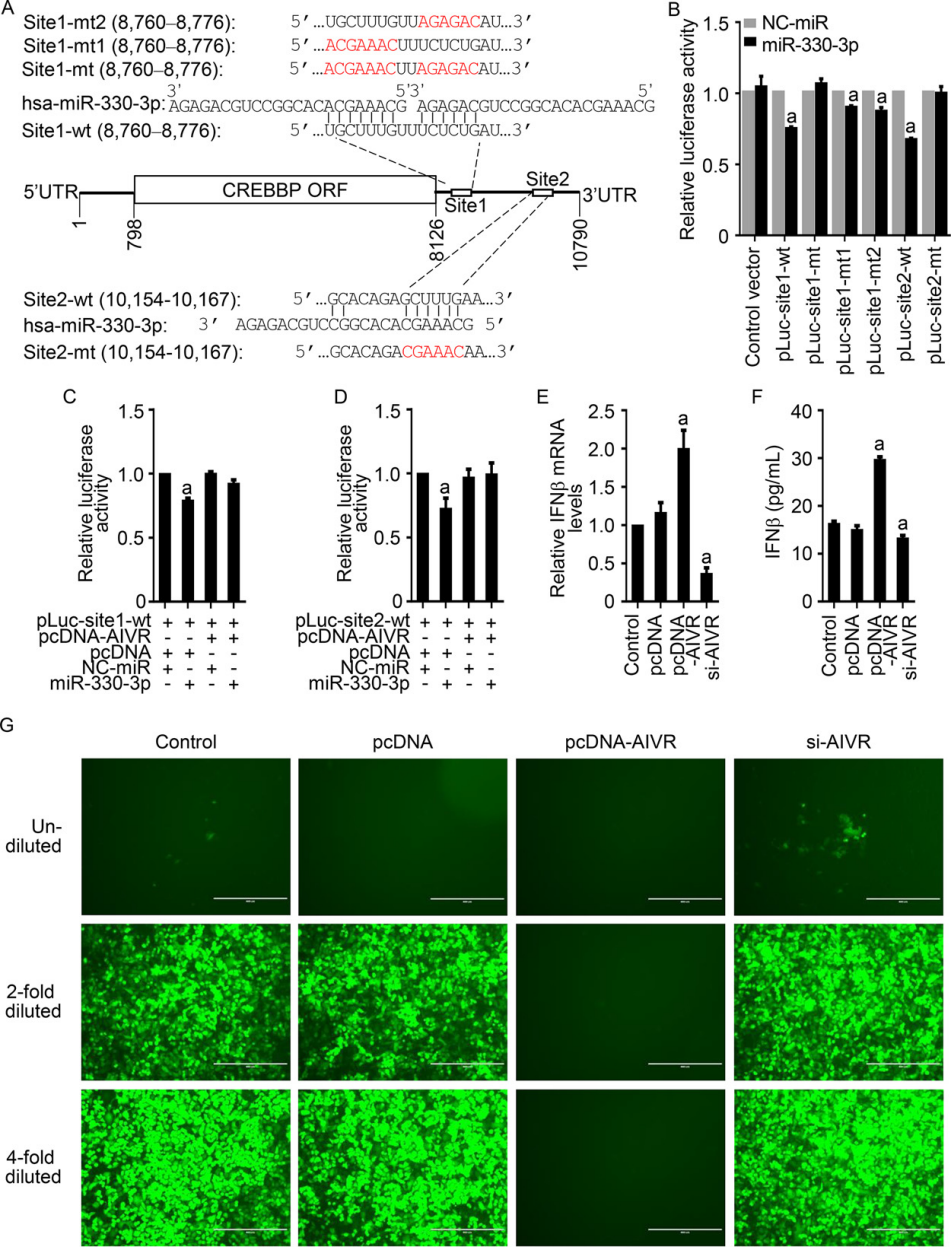
AIVR promotes IFN-β production by absorbing miR-330-3p from CREBBP and then increasing CREBBP expression. (A) Predicted target sites for miR-330-3p in the 3′ UTR of CREBBP. Mutated sequences in the pLuc plasmid used for analysis in B are shown in red. (B) Binding of miR-330-3p to the predicted target sites of CREBBP was evaluated by use of a luciferase reporter gene assay. a, *P *value of <0.01 compared with the corresponding values of the control groups that were transfected with NC-miR. (C and D) AIVR absorbing miR-330-3p from CREBBP site 1 (C) and site 2 (D) was confirmed by use of a luciferase reporter gene assay. a, *P *value of <0.01 compared with the corresponding values of the control groups that were transfected with NC-miR. (E and G) The mRNA levels of IFN-β in AIVR-overexpressing or -silenced A549 cells. A549 cells were untreated or transfected with pcDNA, pcDNA-AIVR, and si-AIVR, and 24 h later, the cells were infected with H7N9 virus. The cellular RNA was collected to examine IFN-β mRNA levels by qRT-PCR (E), and cell supernatants were harvested to detect IFN-β by ELISA (F) or by use of a VSV-GFP report virus bioassay (G). a, *P* value of <0.01 compared with the corresponding values of the control groups that were untreated. Scale bars, 400 μm.

The mRNA of CREBBP has 10,790 nucleotides; its 5′ untranslated region (UTR) has 797 nucleotides, and its 3′ UTR has 2,664 nucleotides. Two sites in the mRNA of CREBBP were predicted to bind to miRNA miR-330-3p by using the TargetScan algorithm (41) (Fig. 7A). Of note, in one of these sites, seven nucleotides match seven nucleotides in the 5′ end of miRNA miR-330-3p, and six nucleotides match six nucleotides in the 3′ end of miRNA miR-330-3p. To verify our prediction, we inserted the sequences bearing these two potential binding sites and their mutants into the pmirGLO luciferase vector and designated the resulting plasmids pLuc-site1-wt, pLuc-site1-mt, pLuc-site1-mt1, pLuc-site1-mt2, pLuc-site2-wt, and pLuc-site2-mt. HEK293T cells were transfected with each of these plasmids and the miR-330-3p or a NC-miR, and the luciferase activities were measured at 36 h after transfection. The luciferase activity in the cells transfected with pLuc-site1-wt, pLuc-site2-wt, pLuc-site1-mt1, or pLuc-site1-mt2 together with miR-330-3p was lower than that in the cells transfected with these plasmids and NC-miR, whereas the luciferase activity in the cells transfected with pLuc-site1-mt or pluc-site2-mt together with miR-330-3p was comparable to that in the cells transfected with these plasmids and the NC-miR (Fig. 7B), indicating that the miRNA miR-330-3p binds to the mRNA of the CREBBP at both predicted sites. We also confirmed that the pLuc-site1-wt- or pLuc-site2-wt-bound miR-330-3p could be absorbed by circRNA AIVR (Fig. 7C and D).

CREBBP is an important component of a large nucleoprotein complex called the IFN-β enhanceosome (42), in which it serves as a molecular connector (43). In virus-infected cells, interferon regulatory factor 3 (IRF-3) and CREBBP form a complex called virus-activated factor, which directly triggers transcription of IFN-β (42, 44). The IRF-3-CREBBP interaction is important for coactivator-mediated localized histone hyperacetylation at the IFN-β promoter (45). We therefore investigated whether overexpression or silencing of circRNA AIVR affected the cellular IFN-β level in influenza virus-infected cells. We found that in AIVR-overexpressing A549 cells, the mRNA and protein levels of IFN-β were significantly higher than those in control cells, and that in AIVR-silenced A549 cells, the mRNA and protein levels of IFN-β were significantly lower than those in control cells (Fig. 7E and F).

Vesicular stomatitis virus (VSV) is very sensitive to IFN-β, and a VSV reporting virus, VSV-green fluorescent protein (GFP), was generated and widely used for evaluating IFN-β levels (10, 46, 47). We therefore further confirmed the above results by using a VSV-GFP system as described previously (47). VSV-GFP did not replicate in the cells treated with the undiluted supernatants that were collected from the control cells and pcDNA-transfected cells, but they replicated well in the cells treated with the diluted supernatants that were collected from these cells. VSV-GFP did not replicate in any cells treated with the supernatants that were collected from the pcDNA-AIVR transfected cells, whereas it replicated in all of the cells treated with supernatants that were collected from the si-AIVR-transfected cells, although its replication in the cells treated with the undiluted supernatants was notably lower than that in other cells (Fig. 7G). These results indicate that IFN-β expression in influenza virus-infected cells was promoted by AIVR overexpression and was inhibited by AIVR silencing.

**FIG 8:**
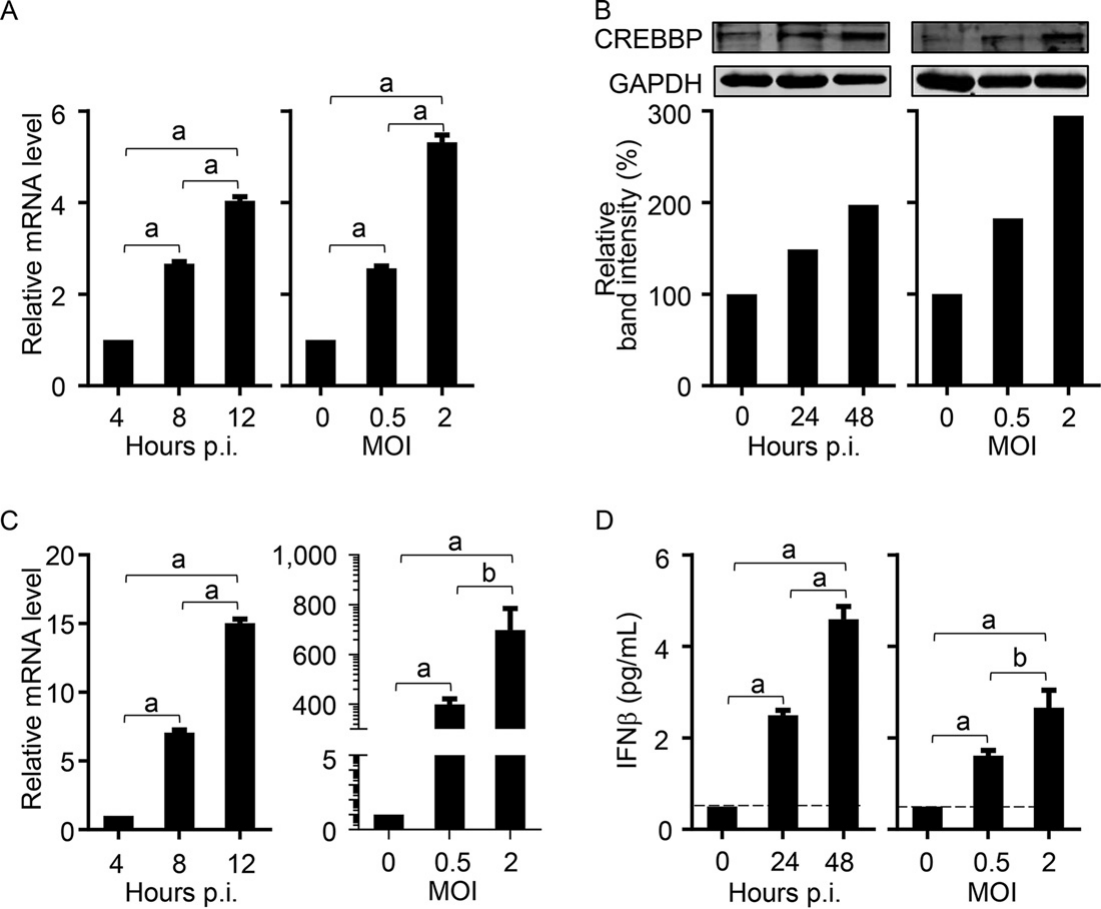
The expression level of CREBBP and IFN-β in influenza virus-infected cells. (A) The mRNA level of the CREBBP in A549 cells infected with H9N2 virus (MOI, 1) at different time points after virus infection (left) or different doses of H9N2 virus at 12 h p.i. (right). (B) Western blotting of the protein level of CREBBP in A549 cells infected with H9N2 virus (MOI, 1) at different time points after virus infection (left) or different doses of H9N2 virus at 24 h p.i. (right). (C) The mRNA level of IFN-β in A549 cells infected with H9N2 virus (MOI, 1) at different time points after virus infection (left) or different doses of H9N2 virus at 12 h p.i. (right). (D) Detection of IFN-β in cell supernatants of A549 cells infected with H9N2 virus (MOI, 1) at different time points (left) or with different doses of H9N2 virus at 24 h p.i. (right) by ELISA. The dashed black lines indicate the lowest limit of detection. a, *P < *0.01. b, *P < 0.05*.

**FIG 9:**
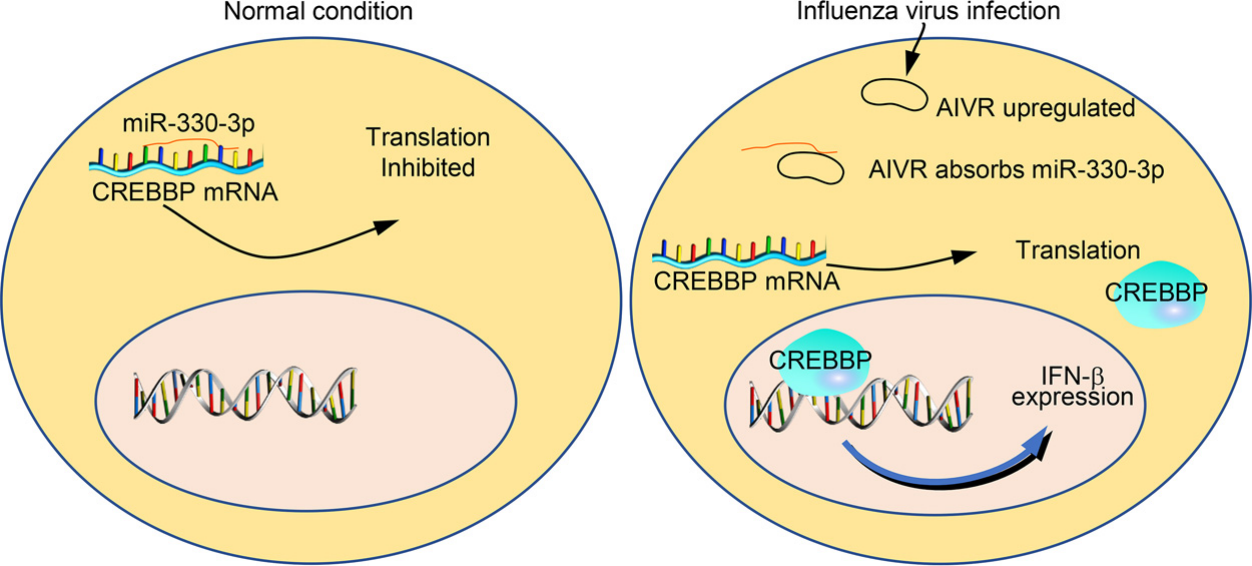
Schematic depiction of the antiviral mechanism involving upregulated circRNA AIVR in influenza virus-infected cells. In normal cells, miRNA miR-330-3p binds to the mRNA of CREBBP and suppresses its replication. In influenza virus-infected cells, the upregulated circRNA AIVR absorbs the miRNA miR-330-3p and thereby promotes CREBBP expression, which then activates IFN-β production.

Our results in Fig. 2 showed that the expression level of AIVR in high-dose virus-infected cells was higher than that in low-dose virus-infected cells, and the expression level of AIVR at 12 h was higher than that at 4 and 8 h (Fig. 2D). We found that the expression levels of CREBBP (Fig. 8A and B) and IFN-β (Fig. 8C and D) in the influenza virus-infected cells were also related to viral dose and infection time, which was consistent with the expression trend of AIVR. Taken together, our findings allow us to present a model of the antiviral mechanism of circRNA AIVR (Fig. 9).

## DISCUSSION

In this study, by using RNA deep sequencing technology and bioinformatics analysis, we detected 11,620 circRNAs in A549 cells and found that 411 of them were differentially expressed upon influenza virus infection. We identified a novel intronic circRNA, AIVR, that was upregulated in influenza virus-infected A549 cells and found that silencing of AIVR significantly promotes influenza virus replication in A549 cells. We found that circRNA AIVR predominantly locates in the cytoplasm and works as an miRNA sponge. Upregulated circRNA AIVR absorbs the miRNA miR-330-3p that binds the mRNA of CREBBP, leading to an increase in the cellular expression of CREBBP, which is an important component of the large nucleoprotein complex IFN-β enhanceosome that accelerates IFN-β production. Therefore, the newly discovered circRNA is an important host innate antiviral factor.

The mechanism of circRNA regulation remains largely unknown. Studies by Rajewsky et al. and Conn et al. reported that the levels of certain circRNAs increase when linear transcript expression also increases but rarely by the same factor (48, 49). Yet some circRNAs show a reciprocal expression pattern during differentiation, suggesting that circularization can compete with linear splicing (50). Chen et al. reported that vesicular stomatitis virus infection of cells led to a decrease in the expression of certain circRNAs, while their cognate mRNAs were largely unaffected (51). Similarly, in this study, we identified 14 intronic circRNAs (5 upregulated and 9 downregulated) that were differentially expressed in influenza virus-infected A549 cells. According to the RNA deep sequencing and bioinformatics analysis, only one of the cognate mRNAs of the five upregulated circRNAs was upregulated in the influenza virus-infected A549 cells, and only two of the cognate mRNAs of the nine downregulated circRNAs were downregulated in the influenza virus-infected A549 cells (see Table S4 in the supplemental material). These facts indicate that the upregulation of circRNA is not necessarily related to the change in their parental genes. Several cellular proteins, including Quaking, MBL, RBM20, NF90/NF110, and ADAR1, have been reported to affect the biogenesis of circRNAs in different cells (50–54). It remains to be investigated which cellular or viral protein(s) promote(s) AIVR upregulation in influenza virus-infected A549 cells.

10.1128/mBio.01017-21.4 TABLE S4 The fold change of differentially expressed intronic circRNAs and their cognate mRNAs. Download Table S4, DOCX file, 0.03 MB. Copyright © 2021 Qu et al. 2021 Qu et al. https://creativecommons.org/licenses/by/4.0/ This content is distributed under the terms of the Creative Commons Attribution 4.0 International license.

Previous studies suggest that circRNAs generated from introns and exons may function differently (55). It has been reported that the intronic circRNAs are abundant in the nucleus and have a positive regulatory effect on their parental coding genes (18, 35, 36), whereas the exonic circRNAs are abundant in the cytoplasm and function as an miRNA sponge (23–26). AIVR is an intronic circRNA that mainly localizes to the cytoplasm and works as a miRNA sponge. Moreover, it does not affect the transcription of its parental gene. These facts suggest that the location and function of circRNAs in cells may not be entirely dependent on their origin and that intronic circRNAs can also localize to the cytoplasm and function as miRNA sponges. AIVR efficiently absorbs six different miRNAs in A549 cells, but only one is associated with influenza virus replication. Although the target mRNAs of the other five miRNAs are unclear, it is reasonable to assume that AIVR has multiple biological functions and affects the mRNAs associated with the other five miRNAs.

In miR-330-3p-transfected cells, the mRNA of CREBBP was degraded, but the mRNA levels of DNAJB1, TNFRSF10D, and PABPN1 increased, suggesting that the miRNA miR-330-3p can interact with multiple mRNAs. Our study demonstrated that miRNA miR-330-3p binds to the 3′ UTR region and suppresses the expression of CREBBP and that this suppression could be partially prevented by the upregulation of AIVR upon influenza virus infection. Previous studies have shown that miRNA can induce gene expression by targeting promoter sequences (21, 22). Target sites of miRNA miR-330-3p were indeed present in the promoters of DNAJB1, TNFRSF10D, and PABPN1 (see Fig. S3 in the supplemental material), which may explain the elevated mRNA levels of these proteins in the cells transfected with miRNA miR-330-3p. However, in A549 cells infected with influenza virus, the upregulation of these three mRNAs may not be due to miRNA miR-330-3p binding to their promoters because our study showed that miRNA miR-330-3p was absorbed by the upregulated circRNA AIVR during influenza virus infection. DNAJB1 and PABPN1 are involved in influenza virus replication at different stages, and TNFRSF10D inhibits apoptosis (56–58); therefore, the upregulation of these proteins in influenza virus-infected cells may be attributable to other as yet unknown causes.

10.1128/mBio.01017-21.7 FIG S3 The target sites of miR-330-3p in the sense strand of promoter DNA of DNAJB1, TNFRSF10D, and PABPN1. The miR-330-3p binding sites in the promoter of DNAJB1, TNFRSF10D, and PABPN1, were predicted by using the online RNAhybrid software (https://bibiserv.cebitec.uni-bielefeld.de/rnahybrid/). Download FIG S3, TIF file, 0.7 MB. Copyright © 2021 Qu et al. 2021 Qu et al. https://creativecommons.org/licenses/by/4.0/ This content is distributed under the terms of the Creative Commons Attribution 4.0 International license.

In summary, we identified a novel intronic circRNA that is upregulated in influenza virus-infected A549 cells, and we further demonstrated that the upregulation of circRNA AIVR is an important part of the host innate IFN-β antiviral pathway. Our study thus provides new insights into the roles of circRNAs in the cellular innate antiviral response.

## MATERIALS AND METHODS

### Viruses.

Influenza viruses A/chicken/Jiangsu/C4258/2012 (H9N2), A/Anhui/1/2005 (H5N1), and A/Anhui/1/2013 (H7N9) were reported previously (59–61) and were propagated in 10-day-old specific-pathogen-free embryonated chicken eggs and stored at −70°C. VSV-GFP was constructed and reported previously (47).

### Cells.

A549 cells, MDCK cells, and HEK293T cells were grown in nutrient mixture F-12 Ham Kaighn’s modified medium or Dulbecco’s modified Eagle’s medium (DMEM) supplemented with 10% fetal bovine serum (Gibco, USA) at 37°C in a humidified 5% CO_2_ atmosphere. Using the Myco-Off mycoplasma cleaner and Myco-Blue mycoplasma detector (Vazyme, China), we ensured that all cells and culture media used in this study were free of mycoplasma contamination.

### Antibodies.

CBP rabbit monoclonal antibody (Cell Signaling Technology, USA) and GAPDH mouse monoclonal antibody (Santa Cruz Biotechnology, USA) were used as the primary antibodies; IRDye800CW goat anti-rabbit IgG (H+L) secondary antibody (Li-Cor, USA) and IRDye800CW goat anti-mouse IgG (H+L) secondary antibody (Li-Cor) were used as secondary antibodies for Western blotting.

### Oligonucleotides.

The sequences for primers and oligonucleotides used in this study are provided in Table S1. The primers for qRT-PCR and plasmid construction were synthesized by TsingKe (Beijing, China). siRNAs, miRNAs, and miRNA inhibitors were synthesized by GenePharma (Shanghai, China). The probes used for FISH and circRNA precipitation were synthesized by General Biol (Anhui, China).

### Plasmid construction.

The circRNA-overexpressing plasmid pcDNA-AIVR was constructed by using the pcDNA3.1 backbone as described previously (62) with minor modifications. Briefly, the full-length linear sequence of AIVR with a 44-nt upstream flanking sequence and an 84-nt downstream flanking sequence was inserted into EcoRV/XhoI sites of pcDNA3.1. Subsequently, the intron 4 fragment of MLLT3/AF9 (chr9, 20414651 to 20415428; hg38) and its inverted fragment were inserted into EcoRI/EcoRV sites and XhoI/XbaI sites, respectively, to facilitate the circularization of the inserted AIVR linear sequence.

For the dual luciferase reporter assay, we constructed eight plasmids bearing different fragments in the pmirGLO dual-luciferase miRNA target expression vector (Promega, USA); the cloning strategy is shown in Fig. 4F. The pLuc-AIVR-wt bears the full-length linear sequence of AIVR. pLuc-site1-wt and pLuc-site2-wt bear a 362-bp fragment and a 413-bp fragment, respectively, that were amplified from the mRNA of CREBBP. pLuc-site1-wt was predicted to contain a canonical site that binds to the 5′ seed region of miR-330-3p and an uncanonical site that binds to the 3′ region of miR-330-3p. The predicted miR-330-3p target sites in the insertions of these plasmids were mutated, and the resulting plasmids were designated pLuc-AIVR-mt, pLuc-site1-mt, pLuc-site1-mt1, pLuc-site1-mt2, and pLuc-site2-mt. The mutated sequences are indicated in Fig. 5A and Fig. 7A.

### Total RNA isolation, library construction, and sequencing.

A549 cells were infected with H9N2 virus at an MOI of 3 for 12 h, and total RNA was extracted from uninfected and H9N2 virus-infected A549 cell samples. After removing the rRNAs, half of the total enriched RNA was used for circRNA and mRNA library construction, and the other half was size selected by polyacrylamide gel electrophoresis to enrich for 18- to 30-nt RNAs for small RNA library construction. To construct circRNA and mRNA libraries, fragmented RNAs were reverse transcribed into cDNA by using random primers, and the cDNA fragments were purified with a QiaQuick PCR extraction kit, end repaired, poly(A) added, and ligated to Illumina sequencing adapters. Then, uracil-*N*-glycosylase was used to digest the second-strand cDNA. The digested products were amplified by PCR and sequenced by Gene Denovo Biotechnology Co. (Guangzhou, China) using an Illumina HiSeq 4000 instrument . To construct the small RNA library, the 3′ and 5′ Illumina sequencing adaptors were ligated to the RNAs, and the ligation products were reverse transcribed, amplified, and sequenced by Gene Denovo Biotechnology Co. using an Illumina HiSeq 2500 instrument.

### qRT-PCR analysis.

Total RNA was extracted using the TRIzol reagent (Invitrogen, USA) according to standard procedures. Reverse transcription of circRNAs and mRNAs was performed by using random primers with a PrimeScript reverse transcriptase (RT) reagent kit with genomic DNA (gDNA) Eraser (TaKaRa, Japan) according to the supplied protocol. Reverse transcription of miRNAs was performed by using our designed stem-loop reverse transcription primers using a PrimeScript RT reagent kit with gDNA Eraser (TaKaRa) or by use of the polyadenylated method using a Mir-X miRNA first-strand synthesis kit (TaKaRa) according to the supplied protocol for a large-scale screen. qRT-PCRs were performed using TB Green Premix *Ex Taq* II (TaKaRa) according to the manufacturer’s instructions. GAPDH was used as an endogenous control for circRNA and mRNA and U6 was used for miRNA. The relative expression was calculated by using the comparative threshold cycle (ΔΔ*CT*) method. The exact expression level of AIVR was calculated by using the standard curve method.

### Cell transfection and infection.

Transfection of siRNAs was conducted by using the Lipofectamine RNAiMax reagent (Invitrogen) at a final concentration of 50 nM according to the manufacturer’s protocol. Transfection of pcDNA-AIVR, miRNA, and miRNA inhibitors was conducted by using Lipofectamine LTX and PLUS reagent (Invitrogen) according to the manufacturer’s protocol. The transfection efficiency was checked by use of qRT-PCR analysis. To investigate the effect of these transfections on the growth of influenza viruses, the above-transfected cells were infected with virus at the indicated MOI for the indicated hours in each experiment. Supernatants were collected, and virus titers were determined in MDCK cells.

### Virus titer determination.

Virus titers were determined in MDCK cells. Briefly, the culture supernatants were serially diluted 10-fold in DMEM with 1 μg/ml tosylsulfonyl phenylalanyl chloromethyl ketone (TPCK)-treated trypsin. A 100-μl aliquot of each diluted sample was added to the well of a 96-well plate containing 70% confluent MDCK cells. Cells were cultured at 37°C in 5% CO_2_ for 72 h. Then, 50 μl of medium per well was used in a hemagglutination assay to identify the presence of virus. We recorded the number of positive and negative wells and calculated the 50% tissue culture infectious dose (TCID_50_) by using the Reed-Muench method.

### Fluorescence *in situ* hybridization (FISH).

AIVR was detected by FISH as described previously with minor modifications (63). Briefly, a 56-nt probe that is complementary to the “head-to-tail” AIVR junction site was synthesized, and 4 thymidine residues of the probe were labeled with cy3. A549 cells seeded on coverslips that were placed in a 12-well plate were transfected with 800 ng of pcDNA-AIVR or infected with H9N2 virus at an MOI of 3. Twenty-four hours posttransfection or 12 h postinfection, the cells (including untreated control cells) were washed twice with phosphate-buffered saline (PBS) and fixed with 1 ml of 4% paraformaldehyde with RNA enzyme inhibitor for 10 minutes at room temperature. The cells were then washed with cold PBS for 10 minutes three times and permeabilized for 2 h in 2 ml of 70% cold ethanol at 4°C. The cells were then incubated at 37°C for 16 h in a solution containing 50% formamide, 10% dextran sulfate, 1% 50× Denhard’s, 10 mM Tris-HCl, 0.3 M NaCl, 1 mM ethylenediaminetetraacetic (EDTA), 100 μg/ml sheared salmon sperm DNA, and 100 ng of FISH probe. They were then washed with 2× saline sodium citrate (SSC) for 15 minutes three times. Then, the cells were incubated with 4,6-diamidino-2-phenylindole (DAPI) for 30 minutes to stain the nuclei and then washed with 2× SSC for 10 minutes three times. Finally, the cells were mounted in ProLong gold antifade (Invitrogen) and were analyzed on a confocal microscope (Zeiss).

### Subcellular fractionation.

A549 cells were seeded in 12-well plates and then transfected with 800 ng of pcDNA-AIVR or were infected with H9N2 virus at an MOI of 0.01. Subcellular fractionation of the control cells, transfected cells, or infected cells was performed by using a PARIS kit (Invitrogen) according to the instructions provided. The cytoplasmic and nuclear RNAs were separately used for qRT-PCR of AIVR. The DNA fragment corresponding to AIVR was amplified and ligated to a T vector, and was used to plot a standard curve by qRT-PCR. AIVR copy numbers in the nucleus or cytoplasm of A549 cells were calculated based on the *C_T_* value from qRT-PCR and the cell number.

### CircRNA precipitation.

CircRNA precipitation was performed as previously reported (64) with minor modifications. A biotin-labeled AIVR probe was designed to be complementary to the head-to-tail junction site. The negative-control biotin-labeled probe was a nonsense sequence. In brief, A549 cells were seeded in a 6-well plate and transfected with pcDNA-AIVR 24 h before use. Cells were washed twice with 2 ml of cold PBS and then lysed by 300 μl of radioimmunoprecipitation assay (RIPA) lysis buffer (Beyotime, China) and 1 μl of RNase inhibitor (TaKaRa, Japan) for 30 minutes on ice and then stored at −80°C. We then ligated the biotin-labeled AIVR probe to Dynabeads magnetic beads (Invitrogen, USA) by following the manufacturer’s instructions. Five microliters of the cell lysate at −80°C was retained as input and 100 μl of lysate supernatant was incubated with the biotin-labeled probe-processed magnetic beads on a rotating wheel at 4°C for 16 h. After the magnetic beads were washed 5 times with cold lysis buffer in a magnetic field, the coprecipitated RNA and 5% input were extracted with TRIzol and detected by qRT-PCR.

### Dual luciferase reporter assay.

HEK293T cells seeded in 24-well plates were collected 36 h after transfection using a luciferase assay system (Promega, USA). In the plasmid and miRNA cotransfection experiment, Lipofectamine LTX and PLUS reagent (Invitrogen, USA) were used according to the manufacturer’s protocol; 100 ng of plasmid was used, and miRNAs were used at a final concentration of 10 nM. The luminescence was measured with a multilabel reader (EnVision, USA). The relative luciferase expression was calculated as the ratio of firefly to Renilla luciferase activity. All experiments were performed in triplicate.

### Western blot analysis.

Western blotting was performed as described previously (11). Briefly, A549 cells seeded in 24-well plates were transfected with miRNA or siRNA or pcDNA-AIVR. Twenty-four hours later, the cells were washed twice with cold PBS and lysed in RIPA lysis buffer (Beyotime, China) containing protease inhibitors (Merck, Germany) and phenylmethylsulfonyl fluoride (PMSF) (Biosharp, China). The protein extracts were denatured at 100°C in SDS-PAGE loading buffer (Beyotime) for 10 minutes. Equal amounts of protein were run on SDS-PAGE. Proteins were transferred onto a nitrocellulose filter membrane (GE Healthcare, USA) and incubated with primary antibody. After being washed with PBS with Tween (PBST), the blots were incubated with secondary antibody and visualized by using an Odyssey CLx infrared imaging system (Li-Cor, USA).

### Detection of IFN-β secretion.

Twenty-four hours after transfection with siRNAs or plasmids, A549 cells were infected with H7N9 virus at an MOI of 0.01. Twenty-four hours later, the cell supernatants were inactivated with UV light for 2 h with constant stirring, and inactivation of the virus was confirmed by cell culture. Detection of IFN-β secretion into cell supernatants was performed by using enzyme-linked immunosorbent assay (ELISA) kits (Invitrogen) following the manufacturer’s instructions. To detect IFN-β secretion by using a bioassay, the UV-inactivated supernatants were serially diluted 2-fold and added to new A549 cells and incubated for 24 h. The cells were then infected with 30 plaque forming units (PFUs) of VSV-GFP for 24 h. VSV-GFP replication was analyzed by fluorescence microscopy.

### Statistical analysis.

Data are presented as mean ± standard deviation (SD) from at least three independent experiments unless otherwise indicated. Comparisons between groups were analyzed by using a two-sided Student's *t* test. A *P* value of <0.05 was considered statistically significant.

### Data availability.

The Gene Expression Omnibus accession number for the circRNA, mRNA, and miRNA sequencing data from this study is GSE172315.
