# Supplementary material for: A Novel Intronic Circular RNA Antagonizes Influenza Virus by Absorbing a microRNA That Degrades CREBBP and Accelerating IFN-β Production
Source: mBio. 2021 Jul 20;12(4):e01017-21. doi: 10.1128/mBio.01017-21 (PMC8406138; doi:10.1128/mBio.01017-21)
Supplement: TABLE S1 [file mbio.01017-21-st001.docx]

**Table S1. Sequences of primers, probes, siRNA, miRNA, and miRNA inhibitors used in this study^a^.**

| Purpose | Primer | Sequence (5'-3') |  |
| --- | --- | --- | --- |
| qRT-PCR | GAPDH-F | GAGTCAACGGATTTGGTCGT |  |
|  | GAPDH-R | GACAAGCTTCCCGTTCTCAG |  |
|  | AIVR-F | GCAATCTACACCCTGGACCT |  |
|  | AIVR-R | CTGTCCCACATGTTGCAGAC |  |
|  | circ_009130-F | GGTTCAGAAATCTCTAGCTCGG |  |
|  | circ_009130-R | GTGTCCCACGAAGCCCCA |  |
|  | circ_009414-F | TGCCATGACCGGAATGTAGT |  |
|  | circ_009414-R | TTGTCAGGATTTCTTTAGCAAA |  |
|  | circ_009456-F | AGTGTTACGGCAACAAGATTA |  |
|  | circ_009456-R | CTTCTGCACCAGCTACCCT |  |
|  | circ_005840-F | CCACGTCCTCGAATTCCCA |  |
|  | circ_005840-R | GGATCTGCCAGTGGTCAAAC |  |
|  | circ_006683-F | CGCAGATACGGGCTTACAGA |  |
|  | circ_006683-R | CATAAAACGTATGAACCAGCAGT |  |
|  | PRKAR1B-mRNA-F | CAGGTCCTCAAAGACTGTATCGT |  |
|  | PRKAR1B-mRNA-R | ATGGGAGTCCGACTGTGAGT |  |
|  | hsa-miR-149-5p-F | TCTGGCTCCGTGTCTTCACTCCC |  |
|  | hsa-miR-7974-F | AGGCTGTGATGCTCTCCTGAGCCC |  |
|  | hsa-miR-3158-3p-F | AAGGGCTTCCTCTCTGCAGGAC |  |
|  | hsa-miR-874-5p-F | CGGCCCCACGCACCAGGGTAAGA |  |
|  | hsa-miR-18a-3p-F | ACTGCCCTAAGTGCTCCTTCTGG |  |
|  | hsa-miR-2682-5p-F | CAGGCAGTGACTGTTCAGACGTC |  |
|  | hsa-miR-330-3p-F | GCAAAGCACACGGCCTGCAGAGA |  |
|  | hsa-miR-1291-F | TGGCCCTGACTGAAGACCAGCAGT |  |
|  | hsa-miR-139-3p-F | TGGAGACGCGGCCCTGTTGGAGT |  |
|  | hsa-miR-3155a-F | CCAGGCTCTGCAGTGGGAACT |  |
|  | hsa-miR-1249-3p-F | ACGCCCTTCCCCCCCTTCTTCA |  |
|  | hsa-miR-3200-3p-F | CACCTTGCGCTACTCAGGTCTG |  |
|  | hsa-miR-188-5p-F | CATCCCTTGCATGGTGGAGGG |  |
|  | hsa-miR-216a-5p-F | TAATCTCAGCTGGCAACTGTGA |  |
|  | hsa-miR-188-3p-F | CTCCCACATGCAGGGTTTGCA |  |
|  | hsa-miR-629-3p-F | GTTCTCCCAACGTAAGCCCAGC |  |
|  | hsa-miR-7976-F | TGCCCTGAGACTTTTGCTC |  |
|  | hsa-miR-486-3p-F | CGGGGCAGCTCAGTACAGGAT |  |
|  | hsa-miR-340-3p-F | TCCGTCTCAGTTACTTTATAGC |  |
|  | hsa-miR-2355-3p-F | ATTGTCCTTGCTGTTTGGAGAT |  |
|  | hsa-miR-1285-5p-F | GATCTCACTTTGTTGCCCAGG |  |
|  | hsa-miR-191-5p-F | CAACGGAATCCCAAAAGCAGCTG |  |
|  | hsa-miR-30b-3p-F | CTGGGAGGTGGATGTTTACTTC |  |
|  | hsa-miR-1285-3p-F | TCTGGGCAACAAAGTGAGACCT |  |
|  | hsa-miR-450a-2-3p-F | ATTGGGGACATTTTGCATTCAT |  |
|  | hsa-miR-34b-5p-F | TAGGCAGTGTCATTAGCTGATTG |  |
|  | hsa-miR-3690-F | ACCTGGACCCAGCGTAGACAAAG |  |
|  | hsa-miR-2355-5p-F | ATCCCCAGATACAATGGACAA |  |
|  | hsa-miR-3144-5p-F | AGGGGACCAAAGAGATATATAG |  |
|  | hsa-miR-221-5p-F | ACCTGGCATACAATGTAGATTT |  |
|  | hsa-miR-3664-3p-F | TCTCAGGAGTAAAGACAGAGTT |  |
|  | hsa-miR-202-5p-F | TTCCTATGCATATACTTCTTTG |  |
|  | hsa-miR-95-3p-F | TTCAACGGGTATTTATTGAGCA |  |
|  | hsa-miR-20a-3p-F | ACTGCATTATGAGCACTTAAAG |  |
|  | hsa-miR-576-3p-F | AAGATGTGGAAAAATTGGAATC |  |
|  | hsa-miR-330-3p-RT | GTCGTATCCAGTGCAGGGTCCGAGGTATTCGCACTGGATAC  GACTCTCTGCAG |  |
|  | hsa-miR-330-3p-F(RT) | CGACGAGCAAAGCACACGGC |  |
|  | hsa-miR-330-3p-R(RT) | CAGTGCAGGGTCCGAGGTAT |  |
|  | U6-RT | CGAGCACAGAATCGCTTCACGAATTTGCGTGTCAT |  |
|  | U6-F | CGAGCACAGAATCGCTTCA |  |
|  | U6-R | CTCGCTTCGGCAGCACATAT |  |
|  | CREBBP-F | CAACCCCAAAAGAGCCAAACT |  |
|  | CREBBP-R | CCTCGTAGAAGCTCCGACAGT |  |
|  | ACTG1-F | CCGAGCCGTGTTTCCTTCC |  |
|  | ACTG1-R | GCCATGCTCAATGGGGTACT |  |
|  | DNAJB1-F | AAGGCATGGACATTGATGACC |  |
|  | DNAJB1-R | GGCCAAAGTTCACGTTGGT |  |
|  | NXT2-F | GGACAAGGCCACCTTAATATGG |  |
|  | NXT2-R | TGGAACTCACTAGAAGGCAATGT |  |
|  | NXF1-F | GACGAGGGGAAGTCGTACAG |  |
|  | NXF1-R | CCAGACCTACGGTTTCCTTCA |  |
|  | TNFRSF10D-F | GTTGGCTTTTCATGTCGGAAGA |  |
|  | TNFRSF10D-R | CCCAGGAACTCGTGAAGGAC |  |
|  | PABPN1-F | TGTGGTTCAGTCAACCGTGT |  |
|  | PABPN1-R | CCTGGTCTGTTGGTTCGTTT |  |
|  | IFNβ-F | ATGACCAACAAGTGTCTCCTCC |  |
|  | IFNβ-R | GCTCATGGAAAGAGCTGTAGTG |  |
| AIVR overexpression plasmid (pcDNA-AIVR) construction | Upstream fragment-F | GGAATTCTTTCTTAATCATCTGAAGCATGGAG |  |
|  | Upstream fragment -R | GATATCTGGACTTAGATTTTGTTCCTAAACA |  |
|  | Downstream fragment-F | GCTCTAGATTTCTTAATCATCTGAAGCATGGAG |  |
|  | Downstream fragment-R | CCGCTCGAGTGGACTTAGATTTTGTTCCTAAACA |  |
|  | Linear AIVR with flanking sequence-F | GATATCTTTTTTGTATTTTTAGTAGAGA |  |
|  | Linear AIVR with flanking sequence-R | CCGCTCGAGGTAGAGATGGGGTTTCACCAT |  |
| AIVR luciferase reporter plasmid (pLuc-AIVR-wt) construction | AIVR linear sequence-F | CCGCTCGAGGCATTTGTTTGACCAGGCACTG |  |
|  | AIVR linear sequence-R | GCTCTAGACATGTTCAGGTTGTGGCTTTC |  |
| AIVR luciferase reporter plasmid (pLuc-AIVR-mt) construction | Mutant overlap primer-F | TGCCCAGCCTGCACACGAAACTTACGGCAGC |  |
|  | Mutant overlap primer-R | GCTGCCGTAAGTTTCGTGTGCAGGCTGGGCA |  |
| CREBBP luciferase reporter plasmid construction (site1 and site2) | pLuc-site1-wt-F | CCGCTCGAGAATGTATCCCGATAACTT |  |
|  | pLuc-site1-wt-R | GCTCTAGATTTCGTATTTATAGGA |  |
|  | pLuc-site2-wt-F | CCGCTCGAGCCAGCACCCAGAGAAC |  |
|  | pLuc-site2-wt-R | GCTCTAGATCCCTACGGGTGGAA |  |
| mutant CREBBP luciferase reporter plasmid construction | pLuc-site1-mt-R1 | TATCAGAGAAAGTTTCGTACAATTTC |  |
|  | pLuc-site1-mt-F1 | GAAATTGTACGAAACTTTCTCTGATA |  |
|  | pLuc-site1-mt-R2 | CCAGTTTTATGTCTCTAAGTTTCGTA |  |
|  | pLuc-site1-mt-F2 | TACGAAACTTAGAGACATAAAACTGG |  |
| pLuc-site2-mt construction | pLuc-site2-mt-R | TATAACTCAAGGTTGTTTCGTCTGTGCTAA |  |
|  | pLuc-site2-mt-F | TTAGCACAGACGAAACAACCTTGAGTTATA |  |
| siRNA | AIVR-siRNA-1 | sense: CAUGGCAUUUGUUUGACCATT |  |
|  |  | antisense: UGGUCAAACAAAUGCCAUGTT |  |
|  | AIVR-siRNA-2 | sense: GAACAUGGCAUUUGUUUGATT |  |
|  |  | antisense: UCAAACAAAUGCCAUGUUCTT |  |
|  | circ_009130-siRNA-1 | sense: UUUGGGGCUUCGUGGGACATT |  |
|  |  | antisense: UGUCCCACGAAGCCCCAAATT |  |
|  | circ_009130-siRNA-2 | sense: CCUUUGGGGCUUCGUGGGATT |  |
|  |  | antisense: UCCCACGAAGCCCCAAAGGTT |  |
|  | circ_009414-siRNA-1 | sense: CUCUUUGCUAAAGAAAUCCTT |  |
|  |  | antisense: GGAUUUCUUUAGCAAAGAGTT |  |
|  | circ_009414-siRNA-2 | sense: UACUCUUUGCUAAAGAAAUTT |  |
|  |  | antisense: AUUUCUUUAGCAAAGAGUATT |  |
|  | circ_009456-siRNA-1 | sense: GUUACGGCAACAAGAUUAATT |  |
|  |  | antisense: UUAAUCUUGUUGCCGUAACTT |  |
|  | circ_009456-siRNA-2 | sense: CAACAAGAUUAACUGCUGATT |  |
|  |  | antisense: UCAGCAGUUAAUCUUGUUGTT |  |
|  | circ_005840 siRNA-1 | sense: GAAUUCCCAGGUGGCCGGCTT |  |
|  |  | antisense: GCCGGCCACCUGGGAAUUCTT |  |
|  | circ_005840 siRNA-2 | sense: CUCGAAUUCCCAGGUGGCCTT |  |
|  |  | antisense: GGCCACCUGGGAAUUCGAGTT |  |
|  | circ_006683-siRNA-1 | sense: UGCUGGUUCAUACGUUUUATT |  |
|  |  | antisense: UAAAACGUAUGAACCAGCATT |  |
|  | circ_006683-siRNA-2 | sense: ACUGCUGGUUCAUACGUUUTT |  |
|  |  | antisense: AAACGUAUGAACCAGCAGUTT |  |
|  | si-NC | sense: UUCUCCGAACGUGUCACGUTT |  |
|  |  | antisense: ACGUGACACGUUCGGAGAATT |  |
| FISH | AIVR-cy3 probe (56 nt) | TTGCAGACAGTGCCTGGTCAAACAAATGCCATGTTCAGGTT  GTGGCTTTCCTTGTT |  |
| circRNA precipitation probe | AIVR-biotin probe | TTGCAGACAGTGCCTGGTCAAACAAATGCCATGTTCAGGTT  GTGGCTTTCCTTGTT-biotin |  |
|  | Control-biotin probe | CTAAGACTAGGTGTTCGGAGGGAAAACAAAAAGAGATATC  AGAA-biotin |  |
| Synthesized miRNAs | miR-330-3p | sense: GCAAAGCACACGGCCUGCAGAGA |  |
|  |  | antisense: UCUCUGCAGGCCGUGUGCUUUGC |  |
|  | hsa-miR-188-3p | sense: CUCCCACAUGCAGGGUUUGCA |  |
|  |  | antisense: UGCAAACCCUGCAUGUGGGAG |  |
|  | hsa-miR-34b-5p | sense: UAGGCAGUGUCAUUAGCUGAUUG |  |
|  |  | antisense: CAAUCAGCUAAUGACACUGCCUA |  |
|  | hsa-miR-95-3p | sense: UUCAACGGGUAUUUAUUGAGCA |  |
|  |  | antisense: UGCUCAAUAAAUACCCGUUGAA |  |
|  | hsa-miR-139-3p | sense: UGGAGACGCGGCCCUGUUGGAGU |  |
|  |  | antisense: ACUCCAACAGGGCCGCGUCUCCA |  |
|  | hsa-miR-149-5p | sense: UCUGGCUCCGUGUCUUCACUCCC |  |
|  |  | antisense: GGGAGUGAAGACACGGAGCCAGA |  |
|  | NC-miR | sense: UUCUCCGAACGUGUCACGUTT |  |
|  |  | antisense: ACGUGACACGUUCGGAGAATT |  |
| Synthesized miRNA inhibitor | miR-330-3p inhibitor | UCUCUGCAGGCCGUGUGCUUUGC |  |
|  | hsa-miR-188-3p inhibitor | UGCAAACCCUGCAUGUGGGAG |  |
|  | hsa-miR-34b-5p inhibitor | CAAUCAGCUAAUGACACUGCCUA |  |
|  | hsa-miR-95-3p inhibitor | UGCUCAAUAAAUACCCGUUGAA |  |
|  | hsa-miR-139-3p inhibitor | ACUCCAACAGGGCCGCGUCUCCA |  |
|  | hsa-miR-149-5p inhibitor | GGGAGUGAAGACACGGAGCCAGA |  |
|  | NC inhibitor | UUCUCCGAACGUGUCACGUTT |  |

a, Mutated sequences are underlined
