# Supplementary figures and images for: A Novel Intronic Circular RNA Antagonizes Influenza Virus by Absorbing a microRNA That Degrades CREBBP and Accelerating IFN-β Production
Source: mBio. 2021 Jul 20;12(4):e01017-21. doi: 10.1128/mBio.01017-21 (PMC8406138; doi:10.1128/mBio.01017-21)

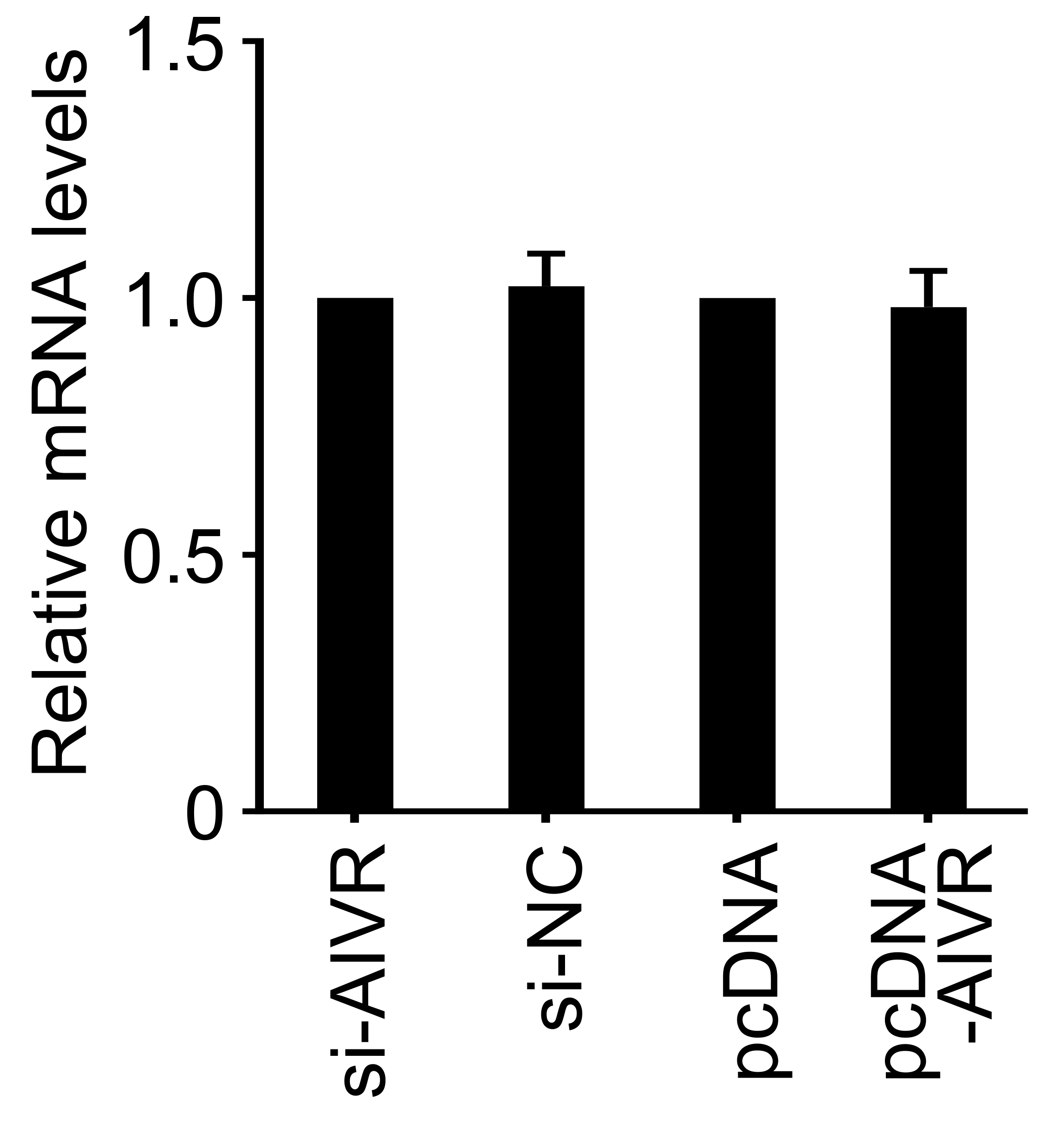

Supplement: FIG S1 [file mbio.01017-21-sf001.tif]

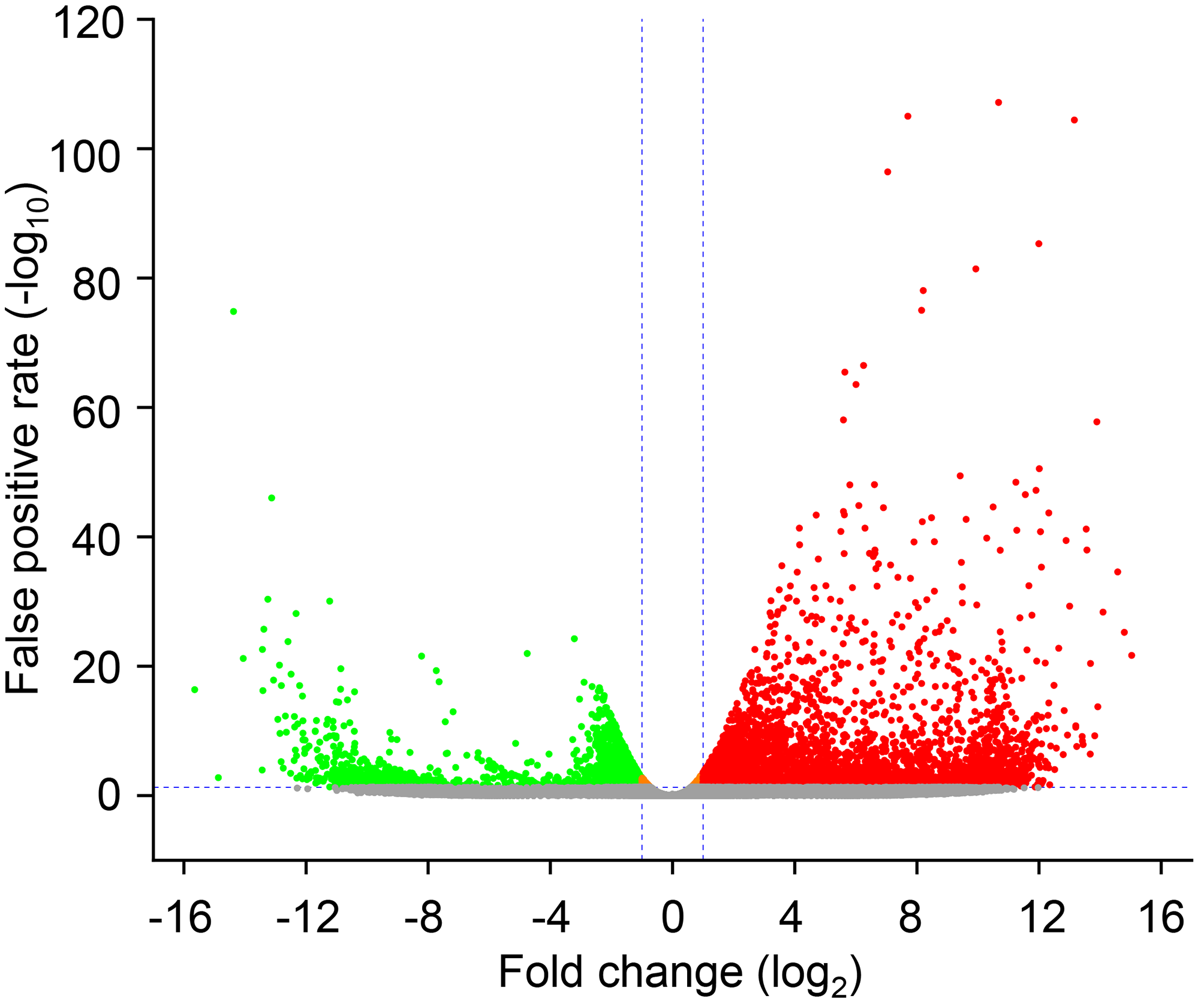

Supplement: FIG S2 [file mbio.01017-21-sf002.tif]

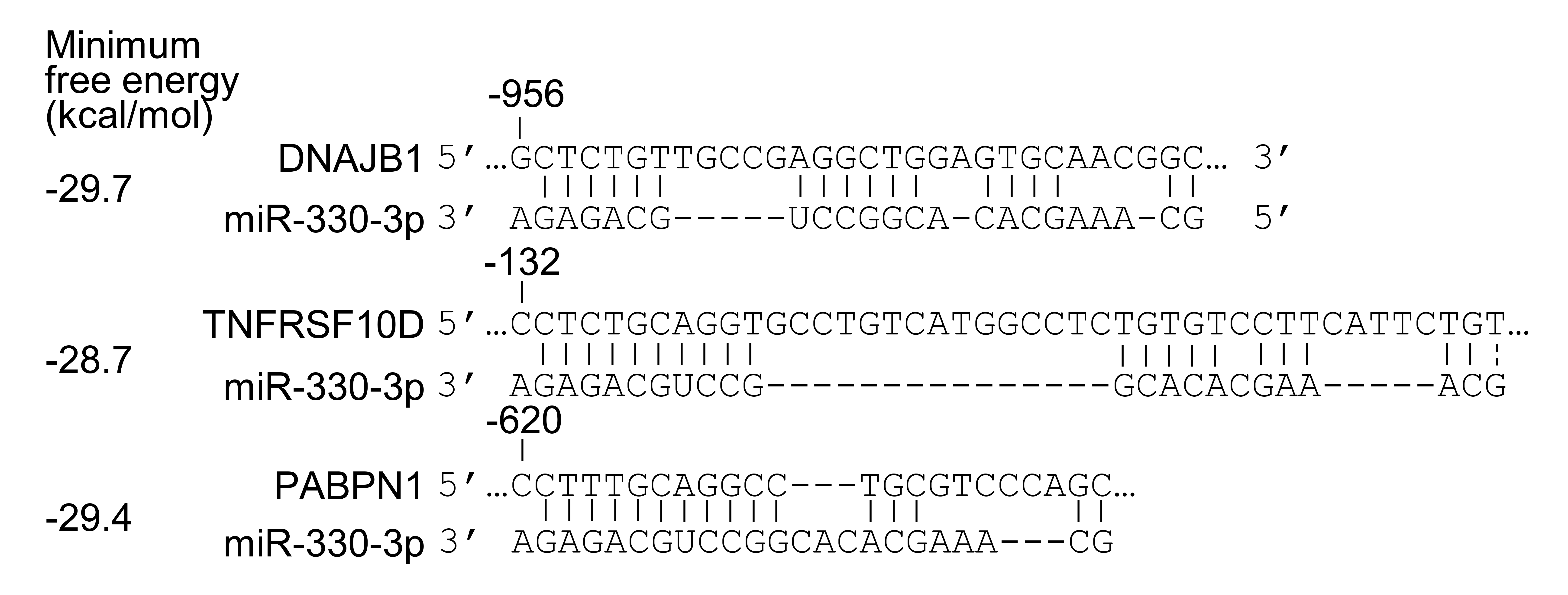

Supplement: FIG S3 [file mbio.01017-21-sf003.tif]
