# Supplementary material for: A Novel Intronic Circular RNA Antagonizes Influenza Virus by Absorbing a microRNA That Degrades CREBBP and Accelerating IFN-β Production
Source: mBio. 2021 Jul 20;12(4):e01017-21. doi: 10.1128/mBio.01017-21 (PMC8406138; doi:10.1128/mBio.01017-21)
Supplement: TABLE S2 [file mbio.01017-21-st002.docx]

**Table S2. The microRNAs (miRNAs) in A549 cells that were predicted to bind circRNA AIVR by RNAhybrid software.**

| miRNA | Minimum free energy (kcal/mol) | Binding position  in AIVR | Average transcripts per million reads in  A549 cells |
| --- | --- | --- | --- |
| hsa-miR-149-5p | -39.2 | 1078 | 301 |
| hsa-miR-7974 | -34.7 | 346 | 188 |
| hsa-miR-3158-3p | -34 | 93 | 34 |
| hsa-miR-874-5p | -32.3 | 1051 | 6 |
| hsa-miR-18a-3p | -32.2 | 1877 | 13 |
| hsa-miR-2682-5p | -32.1 | 1978 | 24 |
| miR-330-3p | -31.6 | 990 | 100 |
| hsa-miR-1291 | -30.8 | 1590 | 7 |
| hsa-miR-139-3p | -30.6 | 1099 | 5 |
| hsa-miR-3155a | -30.5 | 983 | 3.6 |
| hsa-miR-1249-3p | -30.3 | 1083 | 19 |
| hsa-miR-3200-3p | -30 | 1400 | 3.5 |
| hsa-miR-188-5p | -28.6 | 1654 | 5 |
| hsa-miR-216a-5p | -28.3 | 332 | 4 |
| hsa-miR-188-3p | -27.8 | 16 | 7 |
| hsa-miR-629-3p | -27.8 | 644 | 3 |
| hsa-miR-7976 | -27.7 | 691 | 7.9 |
| hsa-miR-486-3p | -27 | 1654 | 133 |
| hsa-miR-340-3p | -26.1 | 1236 | 33 |
| hsa-miR-2355-3p | -26 | 1396 | 10 |
| hsa-miR-1285-5p | -25.9 | 644 | 2.6 |
| hsa-miR-191-5p | -25.4 | 1659 | 23317 |
| hsa-miR-30b-3p | -25.4 | 62 | 4 |
| hsa-miR-1285-3p | -25.4 | 983 | 48 |
| hsa-miR-450a-2-3p | -25 | 1409 | 3 |
| hsa-miR-34b-5p | -24.9 | 771 | 1 |
| hsa-miR-3690 | -24.8 | 1686 | 2.8 |
| hsa-miR-2355-5p | -24.4 | 339 | 251 |
| hsa-miR-3144-5p | -23.3 | 135 | 5 |
| hsa-miR-221-5p | -23.3 | 1080 | 702 |
| hsa-miR-3664-3p | -23.2 | 1539 | 4.6 |
| hsa-miR-202-5p | -21.9 | 1077 | 8.7 |
| hsa-miR-95-3p | -21.5 | 769 | 13 |
| hsa-miR-20a-3p | -21.2 | 898 | 5 |
| hsa-miR-576-3p | -18.6 | 2048 | 11 |
