# Supplementary material for: A Novel Intronic Circular RNA Antagonizes Influenza Virus by Absorbing a microRNA That Degrades CREBBP and Accelerating IFN-β Production
Source: mBio. 2021 Jul 20;12(4):e01017-21. doi: 10.1128/mBio.01017-21 (PMC8406138; doi:10.1128/mBio.01017-21)
Supplement: TABLE S3 [file mbio.01017-21-st003.docx]

**Table S3. Differentially expressed genes of influenza virus infection pathway and their interaction with miRNA miR-330-3p predicted by miRanda.**

| Gene full name (symbol) | Source (access number) | mirSVR  score | phastCons  score |
| --- | --- | --- | --- |
| CREB binding protein (CREBBP) | HGNC (2348) | -0.83 | 0.68 |
| actin gamma 1 (ACTG1) | HGNC (144) | -0.69 | 0.56 |
| DnaJ heat shock protein family (Hsp40) member B1 (DNAJB1) | HGNC (5270) | -0.46 | 0.48 |
| nuclear transport factor 2 like export factor 2 (NXT2) | HGNC (18151) | -0.44 | 0.64 |
| nuclear RNA export factor 1 (NXF1) | HGNC (8071) | -0.32 | 0.69 |
| TNF receptor superfamily member 10d (TNFRSF10D) | HGNC (11907) | -0.11 | 0.52 |
| poly(A) binding protein nuclear 1 (PABPN1) | HGNC (8565) | -0.11 | 0.78 |
| C-X-C motif chemokine ligand 10 (CXCL10) | HGNC (10637) | -0.1 | 0.55 |
| heat shock protein family A (Hsp70) member 6 (HSPA6) | HGNC (5239) | -0.1 | 0.53 |
| C-C motif chemokine ligand 5 (CCL5) | HGNC (10632) | -0.1 | 0.51 |
| tumor necrosis factor (TNF) | HGNC (11892) | -0.01 | 0.66 |
| Fas cell surface death receptor (FAS) | HGNC (11920) | -0.01 | 0.59 |
| tripartite motif containing 25 (TRIM25) | HGNC (12932) | -0.01 | 0.49 |
| signal transducer and activator of transcription 2 (STAT2) | HGNC (11363) | -0.01 | 0.58 |
| PML nuclear body scaffold (PML) | HGNC (9113) | -0.001 | 0.52 |
| cytochrome c, somatic (CYCS) | HGNC (19986) | -0.004 | 0.52 |
| Jun proto-oncogene, AP-1 transcription factor subunit (JUN) | HGNC (6204) | -0.0002 | 0.60 |
| MX dynamin like GTPase 1 (MX1) | HGNC (7532) | No match | No match |
| heterogeneous nuclear ribonucleoprotein U like 1 (HNRNPUL1) | HGNC (17011) | No match | No match |
| radical S-adenosyl methionine domain containing 2 (RSAD2) | HGNC (30908) | No match | No match |
| interferon beta 1 (IFNB1) | HGNC (5434) | No match | No match |
| major histocompatibility complex, class II, DO beta (HLA-DOB) | HGNC (4937) | No match | No match |
| 2’-5’-oligoadenylate synthetase 2 (OAS2) | HGNC (8087) | No match | No match |
| interferon regulatory factor 3 (IRF3) | HGNC (6118) | No match | No match |
| phosphoinositide-3-kinase regulatory subunit 2 (PIK3R2) | HGNC (8980) | No match | No match |
| Fas ligand (FASLG) | HGNC (11936) | No match | No match |
| interferon alpha 17 (IFNA17) | HGNC (5422) | No match | No match |
| interferon alpha 5 (IFNA5) | HGNC (5426) | No match | No match |
| caspase 1 (CASP1) | HGNC (1499) | No match | No match |
| interferon alpha 14 (IFNA14) | HGNC (5420) | No match | No match |
| ribonucleic acid export 1 (RAE1) | HGNC (9828) | No match | No match |
| phosphatidylinositol-4,5-bisphosphate 3-kinase catalytic subunit beta (PIK3CB) | HGNC (8976) | No match | No match |
| interferon regulatory factor 9 (IRF9) | HGNC (6131) | No match | No match |
| interferon induced with helicase C domain 1 (IFIH1) | HGNC (18873) | No match | No match |
| class II major histocompatibility complex transactivator (CIITA) | HGNC (7067) | No match | No match |
| 2’-5’-oligoadenylate synthetase 1 (OAS1) | HGNC (8086) | No match | No match |
| MYD88 innate immune signal transduction adaptor (MYD88) | HGNC (7562) | No match | No match |
| TNF superfamily member 10 (TNFSF10) | HGNC (11925) | No match | No match |
| interferon regulatory factor 7 (IRF7) | HGNC (6122) | No match | No match |
| heat shock protein family A (Hsp70) member 8 (HSPA8) | HGNC (5241) | No match | No match |
| DExD/H-box helicase 58 (DDX58) | HGNC (19102) | No match | No match |
| C-C motif chemokine ligand 2 (CCL2) | HGNC (10618) | No match | No match |
| toll like receptor 3 (TLR3) | HGNC (11849) | No match | No match |
| 2’-5’-oligoadenylate synthetase 3 (OAS3) | HGNC (8088) | No match | No match |
| C-X-C motif chemokine ligand 8 (CXCL8) | HGNC (6025) | No match | No match |
| signal transducer and activator of transcription 1 (STAT1) | HGNC (11362) | No match | No match |
| eukaryotic translation initiation factor 2 alpha kinase 2 (EIF2AK2) | HGNC (9437) | No match | No match |
| NFKB inhibitor alpha (NFKBIA) | HGNC (7797) | No match | No match |
| interferon gamma receptor 2 (IFNGR2) | HGNC (5440) | No match | No match |
| suppressor of cytokine signaling 3 (SOCS3) | HGNC (19391) | No match | No match |
| adenosine deaminase, RNA specific B1 (ADARB1) | HGNC (226) | No match | No match |
| toll like receptor adaptor molecule 1 (TICAM1) | HGNC (18348) | No match | No match |
| heat shock protein family A (Hsp70) member 2 (HSPA2) | HGNC (5235) | No match | No match |
| adenosine deaminase, RNA specific (ADAR) | HGNC (225) | No match | No match |
| Intercellular adhesion molecule 1 (ICAM1) | HGNC (5344) | No match | No match |
| Fas cell surface death receptor (FAS) | HGNC (11920) | No match | No match |
| diphosphoinositol pentakisphosphate kinase 1 (PPIP5K1) | HGNC (29023) | No match | No match |
