# Supplementary material for: A Novel Intronic Circular RNA Antagonizes Influenza Virus by Absorbing a microRNA That Degrades CREBBP and Accelerating IFN-β Production
Source: mBio. 2021 Jul 20;12(4):e01017-21. doi: 10.1128/mBio.01017-21 (PMC8406138; doi:10.1128/mBio.01017-21)
Supplement: TABLE S4 [file mbio.01017-21-st004.docx]

**Table S4. The fold change of differentially expressed intronic circRNAs and their cognate mRNAs.**

| circRNA | Fold change (log _2_) | Cognate mRNA | Fold change (log _2_) |
| --- | --- | --- | --- |
| AIVR | 4.51 | PRKAR1B | No change (/) |
| Novel_circ_002527 | 7.93 | KRT86 | / |
| Novel_circ_006582 | 7.13 | PRM1B | / |
| Novel_circ_011284 | 7.12 | MPD2 | / |
| Novel_circ_006530 | 3.22 | STRN3 | 1.23 |
| Novel_circ_005388 | -2.63 | PMSD12 | / |
| Novel_circ_010363 | -3.29 | TNS3 | -3.31 |
| Novel_circ_010522 | -3.85 | COSTOR3 | / |
| Novel_circ_002854 | -7.6 | MED13L | / |
| Novel_circ_003990 | -7.6 | PKM | 2.24 |
| Novel_circ_010420 | -7.78 | RCC1L | 2.68 |
| Novel_circ_010421 | -7.89 | RCC1L | 2.68 |
| Novel_circ_002708 | -8.08 | TMPO | -1.09 |
| Novel_circ_007333 | -8.43 | ABHD12 | / |
